# Hepatic FoxOs link insulin signaling with plasma lipoprotein metabolism through an apolipoprotein M/sphingosine-1-phosphate pathway

**DOI:** 10.1172/JCI146219

**Published:** 2022-04-01

**Authors:** María Concepción Izquierdo, Niroshan Shanmugarajah, Samuel X. Lee, Michael J. Kraakman, Marit Westerterp, Takumi Kitamoto, Michael Harris, Joshua R. Cook, Galina A. Gusarova, Kendra Zhong, Elijah Marbuary, InSug O-Sullivan, Nikolaus Rasmus, Stefania Camastra, Terry G. Unterman, Ele Ferrannini, Barry E. Hurwitz, Rebecca A. Haeusler

**Affiliations:** 1 Naomi Berrie Diabetes Center,; 2 Department of Pathology and Cell Biology, and; 3 Department of Medicine, Columbia University College of Physicians and Surgeons, New York, New York, USA.; 4 Department of Pediatrics, University Medical Center Groningen, University of Groningen, Groningen, Netherlands.; 5 Division of Endocrinology, Diabetes, and Metabolism, Department of Medicine, University of Illinois, Chicago, Illinois, USA.; 6 Department of Clinical and Experimental Medicine, University of Pisa School of Medicine, Pisa, Italy.; 7 CNR Institute of Clinical Physiology, Pisa, Italy.; 8 Department of Psychology, University of Miami, Coral Gables, Florida, USA.; 9 Behavioral Medicine Research Center and; 10 Division of Endocrinology, Diabetes, and Metabolism, Miller School of Medicine, University of Miami, Miami, Florida, USA.

**Keywords:** Metabolism, Diabetes, Insulin signaling, Lipoproteins

## Abstract

Multiple beneficial cardiovascular effects of HDL depend on sphingosine-1-phosphate (S1P). S1P associates with HDL by binding to apolipoprotein M (ApoM). Insulin resistance is a major driver of dyslipidemia and cardiovascular risk. However, the mechanisms linking alterations in insulin signaling with plasma lipoprotein metabolism are incompletely understood. The insulin-repressible FoxO transcription factors mediate key effects of hepatic insulin action on glucose and lipoprotein metabolism. This work tested whether hepatic insulin signaling regulates HDL-S1P and aimed to identify the underlying molecular mechanisms. We report that insulin-resistant, nondiabetic individuals had decreased HDL-S1P levels, but no change in total plasma S1P. This also occurred in insulin-resistant db/db mice, which had low ApoM and a specific reduction of S1P in the HDL fraction, with no change in total plasma S1P levels. Using mice lacking hepatic FoxOs (L-FoxO1,3,4), we found that hepatic FoxOs were required for ApoM expression. Total plasma S1P levels were similar to those in controls, but S1P was nearly absent from HDL and was instead increased in the lipoprotein-depleted plasma fraction. This phenotype was restored to normal by rescuing ApoM in L-FoxO1,3,4 mice. Our findings show that insulin resistance in humans and mice is associated with decreased HDL-associated S1P. Our study shows that hepatic FoxO transcription factors are regulators of the ApoM/S1P pathway.

## Graphical abstract

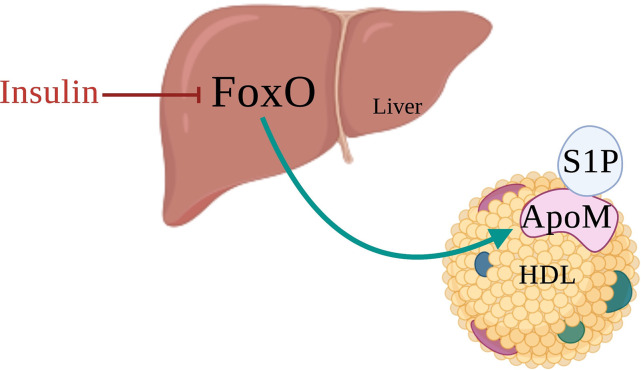

## Introduction

Individuals with insulin resistance and type 2 diabetes have low levels of HDL cholesterol, and low HDL-cholesterol levels are inversely correlated with cardiovascular disease (1). However, clinical trials have demonstrated that raising HDL-cholesterol per se is generally insufficient to reduce coronary disease (2). It is possible that other aspects of HDL are defective in insulin resistance, contributing to cardiovascular risk.

Apolipoprotein M (ApoM) is a secreted protein that is bound to lipoprotein particles and is predominantly enriched — more than 95% — in HDL (3). ApoM is a chaperone for sphingosine-1-phosphate (S1P) in plasma HDL (4). S1P is a bioactive sphingolipid that signals through a series of GPCRs (S1P receptors 1–5) present on a variety of cell types (5, 6). In plasma, approximately 65% of S1P is carried by HDL-bound ApoM, and the remainder is found in the lipoprotein-depleted (LPD) fraction, presumably associated with albumin (4, 7). Of note, S1P induces differential effects, depending on whether it is associated with ApoM or albumin (4, 8, 9). Based on data from *ApoM^–/–^* mice, it has been suggested that ApoM-S1P restrains lymphopoiesis (8) and promotes endothelial barrier function (4, 9, 10). S1P strongly promotes endothelial NOS–dependent (eNOS-dependent) vasodilation by signaling in endothelial cells (11, 12). In vitro, HDL containing S1P enhances eNOS-dependent endothelial barrier activity for a longer time than does albumin containing S1P (13).

Studies in humans have shown that total plasma ApoM levels are reduced in type 2 diabetes (14, 15). Others have demonstrated that total plasma S1P levels are decreased in type 2 diabetes (16). Moreover, total plasma S1P and ApoM levels are inversely associated with mortality in type 2 diabetes (17). However, it is unknown whether the distribution of S1P between different lipoproteins is altered in diabetes pathology. It is also unknown whether this occurs in insulin resistance prior to the onset of type 2 diabetes, when cardiovascular risk is already elevated.

S1P and ApoM have also been investigated in experimental models of insulin resistance and diabetes. Total circulating ApoM levels are decreased in leptin-deficient and leptin receptor–deficient mice (18, 19). On the other hand, inducing hyperglycemia in mice with the β cell toxin streptozotocin is reported to increase total plasma levels of ApoM and S1P (20). Moreover, high-fat diet–induced (HFD-induced) obese mice have been reported to have either reduced plasma ApoM levels (19) or increased plasma ApoM and S1P levels (14). However, the distribution of ApoM and S1P on fractionated lipoproteins has not, to our knowledge, been studied in mouse models of insulin resistance.

Insulin resistance is a major driver of dyslipidemia and cardiovascular risk. However, the mechanisms linking alterations in insulin signaling with dyslipidemia are incompletely understood. The liver is a key tissue for integrating the signaling of insulin and glucose: it is responsible for maintaining the blood glucose concentration (21), and it is a key target for insulin’s inhibition of hepatic glucose production (22). Moreover, the liver is critical to the regulation of lipoproteins: VLDL particles and nascent HDL particles are assembled by the liver. Hepatocytes are the primary site of uptake for LDL particles, and HDL particles also return to the liver to deliver cholesterol removed from other tissues through the process of reverse cholesterol transport (23). Therefore, liver insulin signaling is a potential contributor to insulin resistance–associated lipoprotein abnormalities.

FoxO transcription factors are critical mediators of insulin’s effects on gene expression in hepatocytes, where they are involved in regulating glucose and lipid metabolism (24–28). Indeed, mice with liver-specific knockout of all 3 insulin-sensitive FoxO isoforms (L-FoxO1, -3, and -4, referred to hereafter as L-FoxO1,3,4) have reduced hepatic glucose production and increased de novo lipogenesis (25, 29, 30). These mouse phenotypes are associated with altered expression of genes that regulate glucose versus fatty acid production, including reduced glucose-6-phosphatase catalytic subunit (*G6pc*) and increased glucokinase (25, 29, 31, 32). L-FoxO1,3,4 mice also have defects in bile acid synthesis and HDL-mediated reverse cholesterol transport (33, 34).

In this work, we report that insulin resistance in humans and mice is associated with decreased HDL-associated S1P levels, but not total plasma S1P levels. In mechanistic experiments, we found that the insulin-repressible hepatic FoxO transcription factors promoted hepatic ApoM expression and were required for S1P to associate with HDL particles. This finding suggests a mechanism whereby hepatic insulin signaling via FoxOs determines HDL composition and function.

## Results

### Insulin-resistant individuals have lower HDL-S1P compared with insulin-sensitive individuals.

**Figure 1:**
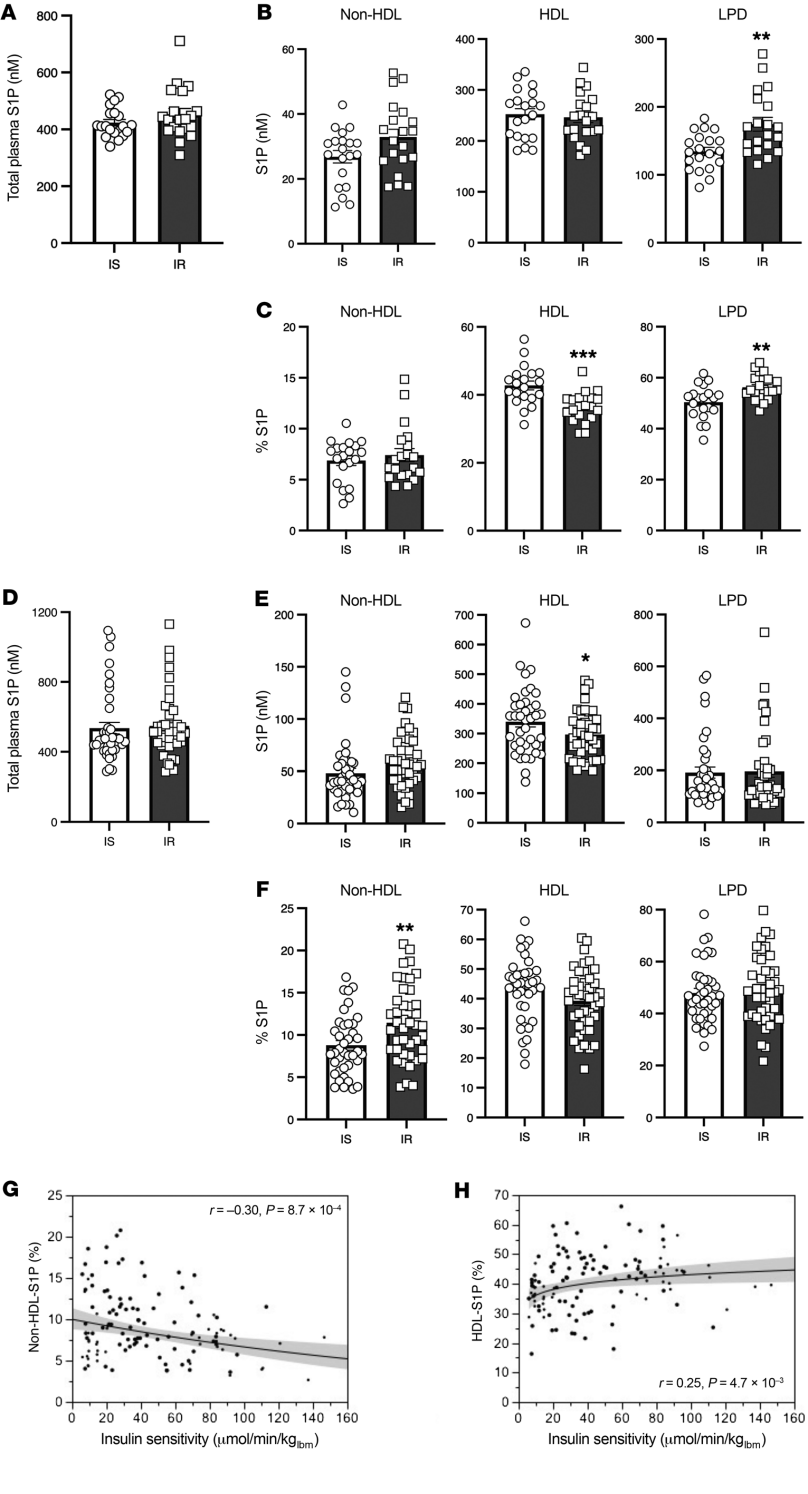
Insulin-resistant individuals have lower HDL-S1P levels than do insulin-sensitive individuals. (**A**–**C**) Cohort 1. (**A**) Total plasma S1P levels. (**B**) Plasma S1P distribution on ultracentrifuge-fractionated lipoproteins. (**C)** Percentage of plasma S1P distribution on lipoproteins. *n =* 20/group for **A**–**C**. (**D**–**F**) Cohort 2. (**D**) Total plasma S1P levels. (**E**) Plasma S1P distribution on ultracentrifuge-fractionated lipoproteins. (**F**) Percentage of plasma S1P distribution on lipoproteins. *n =* 39–42/group for **D**–**F**. (**G** and **H**) Association between insulin sensitivity and S1P content in non-HDL (**G**) and HDL (**H**) particles (expressed as fractions of total plasma S1P). Lines show the nonlinear fit of the data; shaded areas are the 95% CI. lbm, lean body mass. (**G**) log(non–HDL-S1P [%]) = 2.3 – 0.0041 × M. *n =* 121, *r* = 0.30, *P =* 8.7 × 10^–4^. (**H)** log(HDL-S1P [%]) = 3.4 + 0.077 × log(M). *n =* 121, *r* = 0.25, *P =* 4.7 × 10^–3^. Data are presented as the mean ± SEM. *P < 0.05, **P < 0.01, and ***P < 0.001, by Student’s *t* test. IS, insulin-sensitive individuals; IR, insulin-resistant individuals.

We investigated plasma S1P levels and distribution in a cohort of 40 nondiabetic individuals. Of these, 20 individuals were classified as insulin sensitive and 20 as insulin resistant on the basis of the rate of glucose disposal (M value) during hyperinsulinemic-euglycemic clamps. The clinical and metabolic characteristics of the cohort are summarized in Supplemental Table 1; supplemental material available online with this article; https://doi.org/10.1172/JCI146219DS1 We fractionated lipoproteins by sequential density ultracentrifugation and measured S1P levels in total and fractionated plasma by liquid chromatography–mass spectrometry (LC-MS). We detected no differences in total plasma S1P levels between the groups (Figure 1A). In fractionated plasma, insulin-resistant individuals showed a slightly higher concentration of S1P in the LPD fraction, balanced by a trend toward a slightly lower S1P concentration in the HDL fraction (Figure 1B). Because there was no difference in total plasma S1P levels, we also calculated the distribution of S1P as a percentage of the total. Insulin-resistant individuals had a significant reduction of S1P in the HDL fraction and an increase of S1P in the LPD fraction compared with insulin-sensitive individuals (Figure 1C). We note that it is not yet established whether the absolute values of HDL-S1P concentration or the distribution of S1P on HDL versus LPD is more biologically relevant. The levels of other sphingolipids measured are reported in Supplemental Table 3.

Next, we examined an independent cohort of 81 individuals to validate the findings described above. Thirty-nine of these individuals were classified as insulin sensitive and 42 were classified as insulin resistant on the basis of the clamp-derived glucose disposal rates. The clinical and metabolic characteristics of the cohort are summarized in Supplemental Table 2. We noted no differences in total plasma S1P levels (Figure 1D). However, insulin-resistant individuals showed a reduction of S1P in the HDL fraction compared with levels in the insulin-sensitive individuals (Figure 1E). When the distribution was expressed as a percentage of the total, the trend was the same (Figure 1F). Of note, in cohort 2, we did not observe a significant redistribution of S1P to the LPD fraction, although there was an increase in the percentage of S1P on non-HDL. Thus, although the relative depletion of S1P from HDL was consistent across both cohorts, the redistribution of S1P to other fractions varied. The levels of other sphingolipids measured are reported in Supplemental Table 4.

In the pooled data from both cohorts (*n =* 121), HDL-S1P was directly, and non–HDL-S1P was inversely, related to the M value (Figure 1, G and H); however, there was no relationship with LPD S1P (data not shown). Neither association was significantly altered by adjustment for sex, BMI, or both. When the above 2 models were adjusted for HDL concentrations in a bivariate regression, the independent association of non–HDL-S1P (percentage) with M became borderline (*P =* 0.07), whereas the independent association of HDL-S1P (percentage) with M retained full statistical significance (*P <* 0.01). Taken together, these results indicate that S1P distribution onto HDL was reduced in insulin-resistant, nondiabetic individuals in 2 independent cohorts with different ethnicities.

HDL-S1P has been suggested to promote vascular endothelial function, including vasodilation (4, 13, 35, 36). Moreover, insulin resistance is known to impair flow-mediated dilation (37). Therefore, we reasoned that the reductions in HDL-S1P in insulin-resistant participants could be involved in their impaired flow-mediated dilation. However, we found no correlation between HDL-S1P and flow-mediated dilation (median [IQR] values of flow-mediated dilation in insulin-sensitive and insulin-resistant participants were 5.1% [7.6%] and 3.9% [3.1%], *P =* 0.14 by Mann-Whitney *U* test and for the linear correlation of HDL-S1P with flow-mediated dilation [FMD], *r* = 0.15 *P =* 0.17).

### ApoM expression and HDL-associated S1P levels are decreased in db/db, but not diet-induced obese mice.

**Figure 2:**
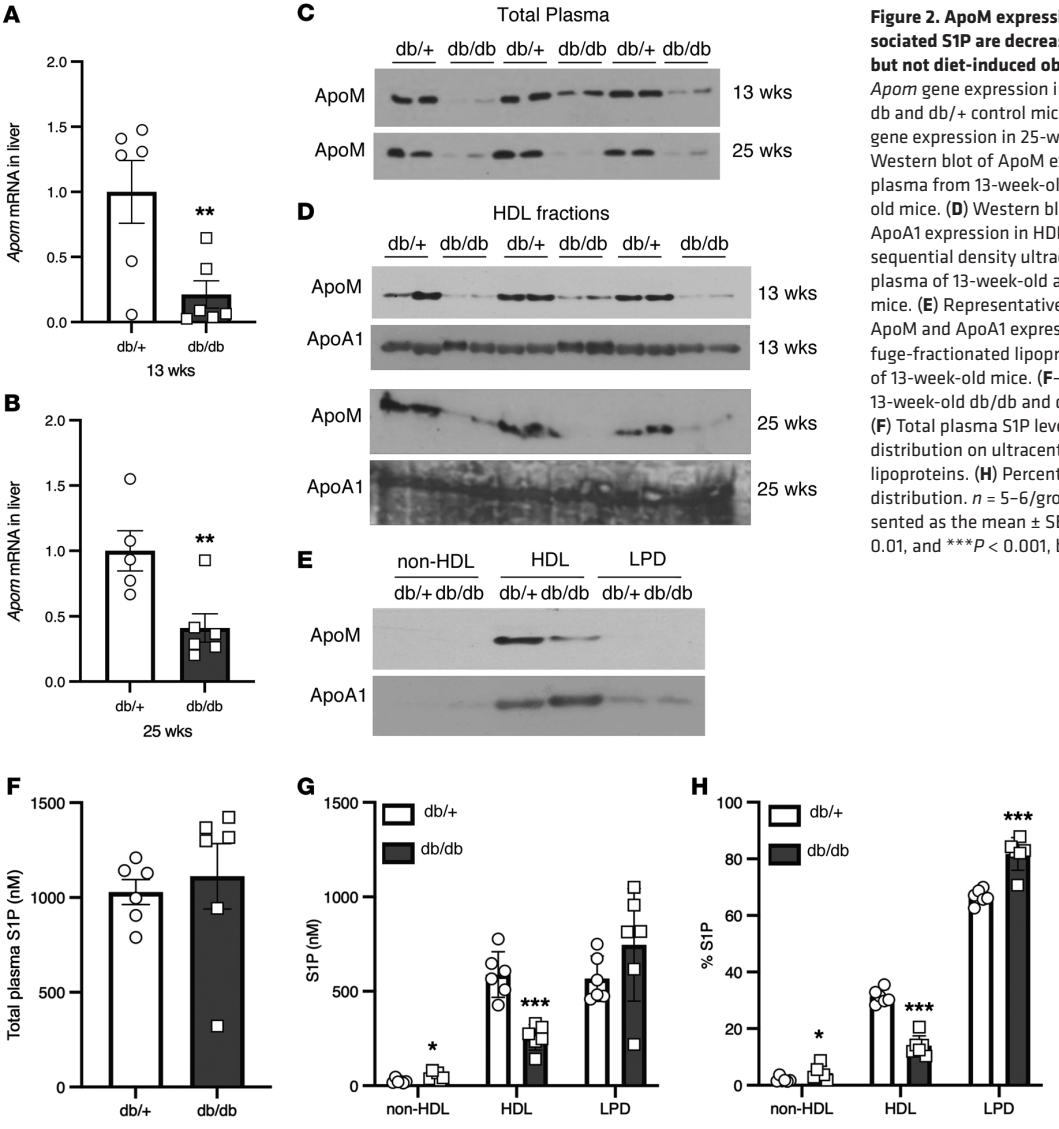
ApoM expression and HDL-associated S1P are decreased in db/db mice but not diet-induced obese mice. (**A**) Liver *Apom* gene expression in 13-week-old db/db and db/+ control mice. (**B**) Liver *Apom* gene expression in 25-week-old mice. (**C**) Western blot of ApoM expression in total plasma from 13-week-old and 25-week-old mice. (**D**) Western blot of ApoM and ApoA1 expression in HDL fractionated by sequential density ultracentrifugation from plasma of 13-week-old and 25-week-old mice. (**E**) Representative Western blot of ApoM and ApoA1 expression in ultracentrifuge-fractionated lipoproteins from plasma of 13-week-old mice. (**F**–**H**) S1P levels in 13-week-old db/db and db/+ control mice. (**F**) Total plasma S1P levels. (**G**) Plasma S1P distribution on ultracentrifuge-fractionated lipoproteins. (**H**) Percentage of plasma S1P distribution. *n =* 5–6/group. Data are presented as the mean ± SEM. *P < 0.05, **P < 0.01, and ***P < 0.001, by Student’s *t* test.

We investigated ApoM expression and S1P distribution in mouse models of hyperinsulinemia and insulin resistance. We examined leptin receptor–deficient db/db mice at 2 different ages: 13 and 25 weeks. The body weight, glucose, and insulin levels were higher in the db/db mice, as expected (Supplemental Figure 1, A–C). We observed that hepatic *Apom* expression was reduced in db/db mice (Figure 2, A and B). Consistent with this, ApoM protein levels were reduced in total plasma (Figure 2C) and specifically in the HDL fractions from db/db mice (Figure 2, D and E). We detected no differences in total plasma S1P levels (Figure 2F), suggesting that there were no defects in the generation and secretion of S1P into plasma. However, the db/db mice showed a reduction of S1P in HDL and an increase of S1P in the LDP and non-HDL fractions (Figure 2, G and H). The levels of other sphingolipids measured are reported in Supplemental Table 5. These findings show that transcriptional regulation of endogenous hepatic *Apom* was sufficient to modulate HDL-S1P content and that this regulation was altered in db/db mice.

Next, we examined diet-induced obese mice. We fed C57BL/6J mice an obesogenic diet starting at 6 weeks of age, until the harvesting of tissues, when the mice were 13 or 29 weeks old. We observed an increase in body weight, glucose, and insulin levels in the diet-induced obese mice, as expected (Supplemental Figure 2, A–C). However, we observed no difference in ApoM mRNA or protein expression (Supplemental Figure 2, D–F). Total plasma S1P levels were higher in the diet-induced obese mice, with no differential distribution on lipoproteins (Supplemental Figure 2, G–I). The levels of other sphingolipids measured are reported in Supplemental Table 6. Altogether, these data suggest that ApoM expression and S1P binding to HDL are affected in some, but not all, models of obesity, hyperinsulinemia, and insulin resistance.

### FoxOs are required for hepatic ApoM expression.

We next investigated potential mediators of the reduced ApoM-S1P levels observed in the above experiments. FoxO transcription factors are key regulators of hepatic insulin action. It is widely held that FoxOs are constitutively active in insulin resistance, due to impaired Akt-mediated phosphorylation (38). On the other hand, it has also been suggested that the absence of FoxOs may mimic some conditions of hyperinsulinemia (25, 39), because FoxOs are exquisitely sensitive to very low levels of insulin (40). FoxOs can also be acetylated and rapidly degraded in response to hyperglycemia and other oxidative stresses (41). So, we explored the role of hepatic FoxOs in ApoM expression.

**Figure 3:**
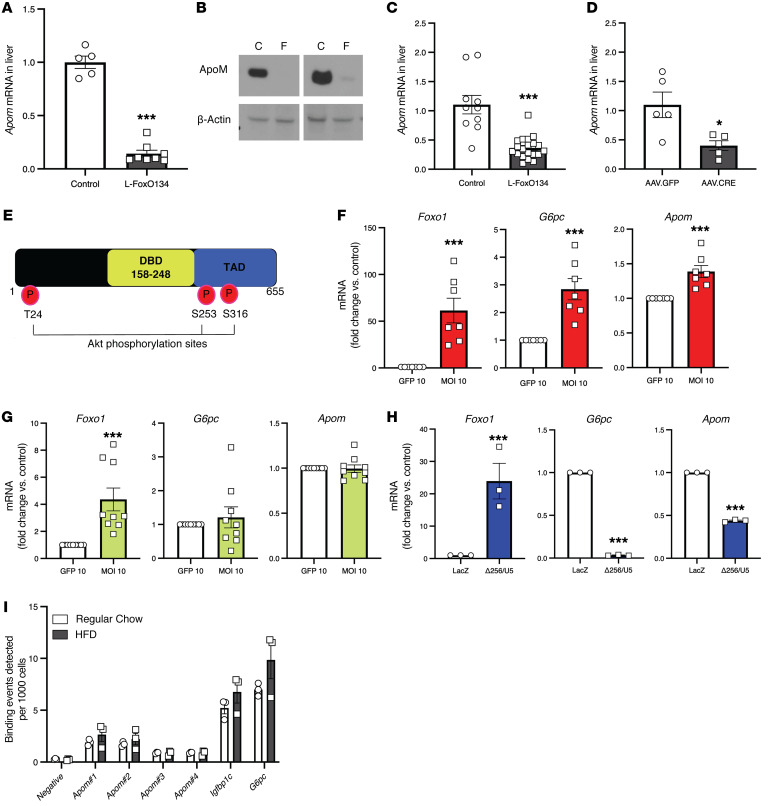
ApoM is a transcriptional target of FoxO. (**A**) Hepatic *Apom* gene expression in adult male mice (*n =* 5–8/group). (**B**) Representative Western blots of ApoM expression in liver lysates from adult male mice. C, littermate control mice; F, L-FoxO1,3,4 mice. (**C**) Hepatic *Apom* gene expression in mice of both sexes that were sacrificed on P2 (*n =* 10–20/group). (**D**) Hepatic *Apom* gene expression following acute knockdown via AAV8.Tbg.Cre in adult male *Foxo1^fl/fl^*, *Foxo3^fl/fl^*, and *Foxo4^fl/Y^* mice (*n* = 5/group). Values are shown relative to littermate controls. (**E**) Schematic representation of FoxO1 protein. (**F**–**H**) *Foxo1*, *G6pc*, and *Apom* gene expression in primary hepatocytes from WT mice that were transduced with different FoxO1 mutants. (**F**) FoxO1-ADA mutant: the 3 Akt phosphorylation sites are mutated, causing FoxO1 to be constitutively nuclear. Data indicate the mean ± SEM of triplicates of 3 independent experiments. (**G**) FoxO1-ADA-DBD mutant: contains the ADA mutation and a mutation disrupting the DNA binding domain. Data indicate the mean ± SEM of triplicates of 3 independent experiments. (**H**) FoxO1-Δ256 mutant: a dominant-negative version of FoxO1 that lacks the transactivation domain. (**I**) Chip-qPCR of *Apom*, *Igfbp1*, and *G6pc* from livers of mice with a knockin allele of FoxO1-Venus. The mice were fed either chow or a HFD for 4 weeks. Data are presented as the mean ± SEM. *P < 0.05 and ***P < 0.001, by Student’s *t* test.

To examine the effects of hepatic FoxO deletion on apolipoprotein gene expression, we queried our microarrays from prior experiments (25). We found that livers from L-FoxO1,3,4 mice had a greater than 80% reduction in the mRNA expression of *Apom* compared with littermate controls (*P =* 0.006) (25). To confirm this, we carried out quantitative PCR (qPCR) and Western blot analyses in liver tissue and found that L-FoxO1,3,4 mice had approximately 90% reductions in *Apom* mRNA expression and nearly undetectable ApoM protein levels in liver (Figure 3, A and B).

The effect of FoxO deletion on reducing hepatic *Apom* expression might have been due to a primary effect of FoxO ablation or an acquired or compensatory defect due to long-term genetic loss of FoxOs. Thus, we measured hepatic *Apom* expression levels in neonatal mice. We detected decreases in *Apom* as early as P2 in neonatal L-FoxO1,3,4 mice compared with expression in littermate controls (Figure 3C). To test the effect in adult mice, we examined mice with an acute depletion of FoxOs. We transduced adult mice bearing *Foxo1^fl/fl^*, *Foxo3^fl/fl^*, and *Foxo4^fl/Y^* alleles with an adeno-associated virus expressing Cre recombinase under the hepatocyte-specific Tbg promoter (AAV8.Tbg.Cre). We found that 1 month after the injection, there was a greater than 80% decrease in *Foxo* mRNA expression as well as low levels of *G6pc*, a known target of FoxO transcriptional activation (34). In these FoxO-depleted mice, we observed that *Apom* mRNA expression was significantly decreased (Figure 3D). These findings demonstrate that hepatic FoxOs are required for hepatic *Apom* expression.

### ApoM is a transcriptional target of FoxO.

We next examined whether hepatic FoxO activity is sufficient for hepatic *Apom* expression. Zhang and colleagues generated a transgenic mouse line containing the human FoxO1 gene with mutations in the 3 Akt phosphorylation sites, thus blocking FoxO1’s nuclear exclusion, under the control of the hepatocyte-selective α-1-antitrypsin promoter (42). In microarray experiments, the transgenic mice showed a significant 77% increase in liver *Apom* expression. To support this finding with qPCR, we obtained liver tissue from a small number of these transgenic mice and their control littermates and detected a 63% increase in *Apom* expression (Supplemental Figure 3), consistent with the published finding.

Hepatic FoxOs have been suggested to modulate some liver metabolic pathways via indirect effects on nonhepatic tissues (43, 44). Therefore, we examined whether FoxOs regulate *Apom* cell autonomously in primary hepatocytes using mutant versions of FoxO1. The FoxO1 protein structure contains 3 Akt phosphorylation sites that mediate its nuclear exclusion, a transactivation domain, and a DNA binding domain (Figure 3E and refs. 45, 46). We isolated primary hepatocytes from WT mice and transduced them with a FoxO1-ADA mutant, which has mutations in the 3 Akt phosphorylation sites, causing FoxO1 to be constitutively nuclear (47). The FoxO1-ADA mutant increased the expression of *G6pc* — a canonical FoxO target — and *Apom* (Figure 3F). FoxOs can regulate gene expression by direct DNA binding or by acting as a transcriptional coregulator (48–51). Thus, we evaluated whether the DNA binding domain of FoxO1 is required for the induction of *Apom*. We used a FoxO1-ADA-DBD mutant, which has the ADA mutation as well as a second mutation in the DNA binding domain. We observed that the ADA-DBD mutant of FoxO1 was unable to activate *G6pc* or *Apom* expression (Figure 3G). We also transduced primary hepatocytes with a dominant-negative version of FoxO1 that lacks the transactivation domain (FoxO1-Δ256; ref. 52). We found that FoxO1-Δ256 decreased *G6pc* and *Apom* expression (Figure 3H). These data indicate that FoxOs promoted hepatic *Apom* expression by direct actions in hepatocytes and that the DNA binding and transactivation domains were required.

To determine whether FoxO1 binds to the promoter and enhancer regions of *Apom*, we performed ChIPs on liver from mice, fed chow or a HFD for 4 weeks, that had a knockin allele of FoxO1-Venus (53). We found that FoxO1 bound to 2 regions of the *Apom* promoter and, to a lesser extent, 2 regions of the *Apom* enhancer (Figure 3I). Notably, there were no differences between chow- and HFD-fed mice in FoxO1 occupancy at the promoters of *Apom*, *G6pc*, or *Igfbp1* (Figure 3I).

### A subset of FoxO transcriptional complexes may be inactivated in db/db mice.

Based on the widely held notion that FoxOs are constitutively active in insulin resistance, it might be expected that FoxOs would be constitutively activated in db/db mice. To test this, we investigated the expression of other known FoxO targets in these db/db mice. We observed that *G6pc* was not elevated in the db/db mice at either age, although *Igfbp1* was (Supplemental Figure 1D). *Gck* is normally suppressed by FoxOs (25, 32), but it was increased in the 13-week-old db/db mice (Supplemental Figure 1D). Therefore, these canonical FoxO targets in the glucose metabolism pathway were differentially consistent with FoxO being active, inactive, or unaffected in db/db mice, and there was no clear across-the-board induction of FoxO targets that would indicate constitutive FoxO activation. On the other hand, we have previously reported that *Scarb1* and *Lipc*, 2 hepatic genes involved in HDL cholesterol uptake into the liver, are induced by FoxOs (34). Here, we found that both *Scarb1* and *Lipc* were reduced in db/db mice (at 25 weeks and at both ages, for *Scarb1* and *Lipc*, respectively) (Supplemental Figure 1E). These findings suggest the possibility that in db/db mice, a subset of FoxO target genes related to lipoprotein metabolism (i.e., *Apom*, *Scarb1*, and *Lipc*) were inactivated through an unknown mechanism.

### Hypothalamic obesity caused by gold thioglucose injury decreases Apom in a partially FoxO-dependent manner.

**Figure 4:**
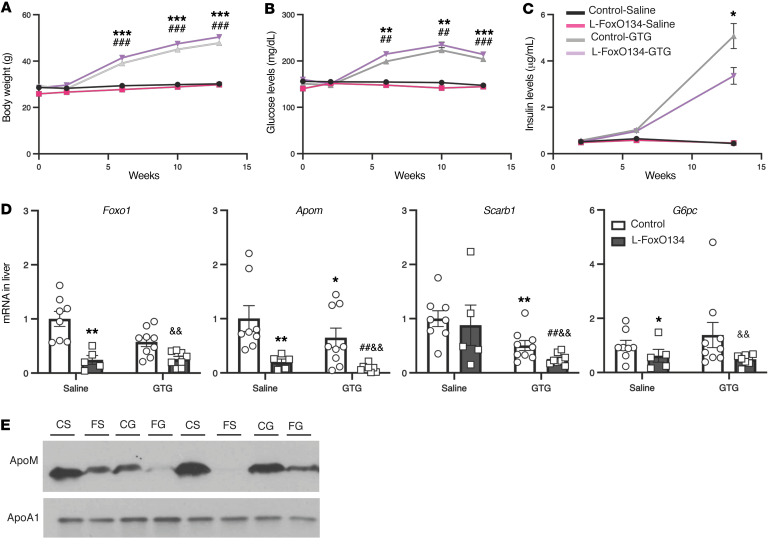
Hypothalamic obesity caused by gold thioglucose injury reduces *Apom* in a partially FoxO-dependent manner. Chow-fed mice were injected intraperitoneally with gold-thioglucose or saline and were continued on a chow diet for 13 weeks. (**A**) Total body weight. (**B**) Plasma glucose levels after 5 hours fasting. (**C**) Plasma insulin levels after 5 hours fasting. (**D**) Hepatic *Foxo1*, *Apom*, *Scarb1*, and *G6pc* gene expression. (**E**) Representative Western blot of ApoM and ApoA1 expression in total plasma from chow-fed mice. CS, littermate control mice treated with saline; FS, L-FoxO1,3,4 mice treated with saline; CG, littermate control mice treated with gold-thioglucose; FG, L-FoxO1,3,4 mice treated with gold-thioglucose. *P < 0.05, **P < 0.01, ***P < 0.001 versus control saline. ^##^*P <* 0.01 and ^###^*P <* 0.001 versus L-FoxO134-saline. ^&&^*P <* 0.01 versus control-GTG, by 2-way ANOVA. Data are presented as the mean ± SEM. (*n =* 5–9/group for all panels)

Next we aimed to determine whether the effects of the db/db mutation on *Apom* occur in other forms of hypothalamic obesity and whether the effects are mediated by hepatic FoxOs. To do so, we performed experiments using gold thioglucose, which induces hypothalamic lesions and hyperphagia (54). We injected adult male control and L-FoxO1,3,4 mice with 0.6 g/kg gold thioglucose or an equivalent volume of saline and continued feeding the mice chow for 13 weeks. In the gold thioglucose–injured mice of both genotypes, body weight, fasting blood glucose, and fasting plasma insulin all increased substantially (Figure 4, A–C). In control mice, gold thioglucose injury caused a significant decrease in hepatic *Apom* expression (Figure 4D). In L-FoxO1,3,4 mice, the nonobese mice already showed low *Apom* expression, and *Apom* levels were slightly decreased even further after gold thioglucose injury. We confirmed these changes in circulating ApoM by Western blotting (Figure 4E). Of interest, *Scarb1* showed a similar pattern of decreased expression in the gold thioglucose–injured mice, but *G6pc* was reduced in L-FoxO1,3,4 mice and unaffected by gold thioglucose (Figure 4D). Taken together with our findings in db/db mice, these data are consistent with an effect of hypothalamic obesity causing decreased ApoM through a hepatic FoxO–dependent mechanism and also, to a lesser extent, a FoxO-independent mechanism.

### FoxOs are required for S1P binding to HDL.

**Figure 5:**
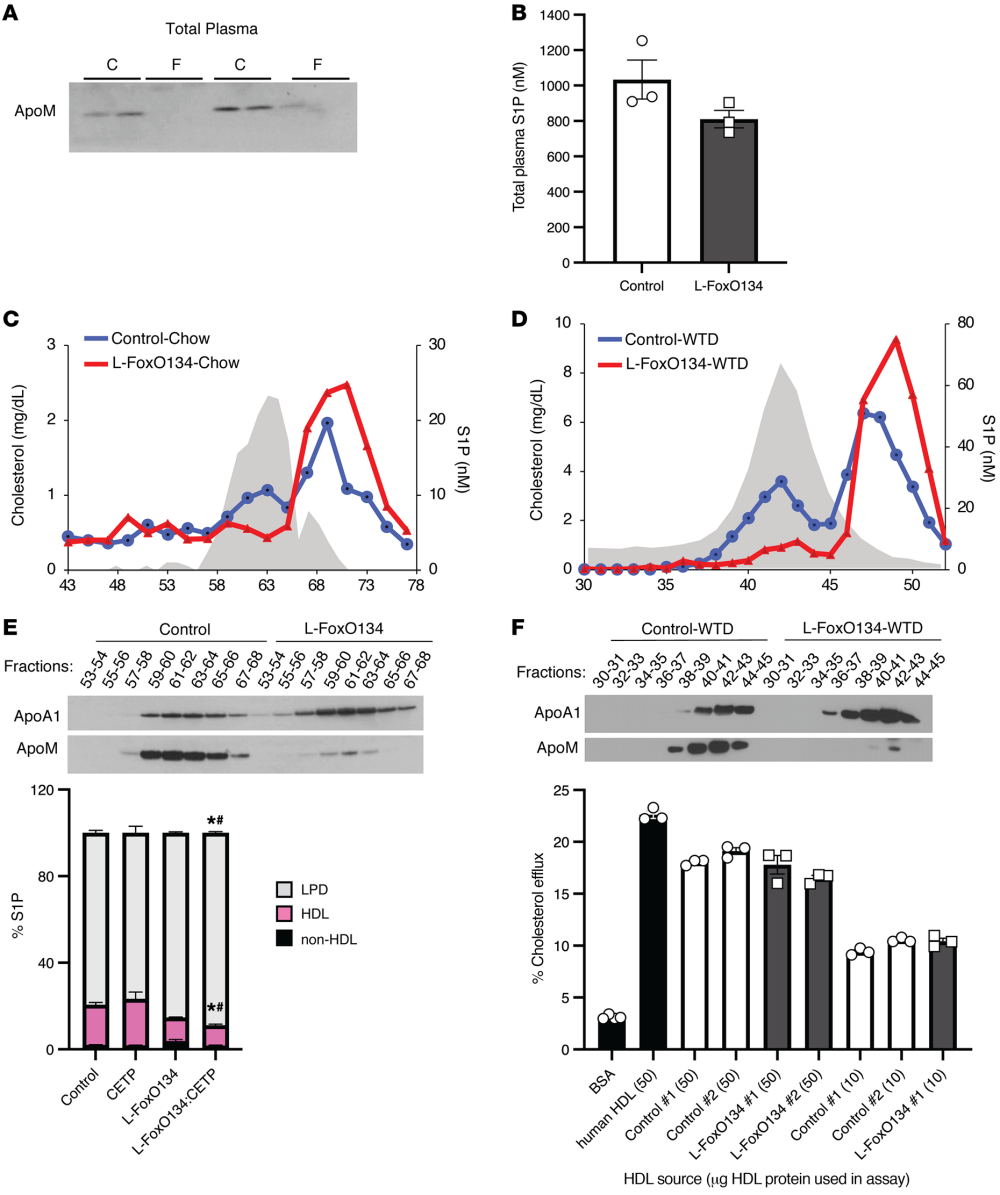
FoxOs are required for binding of S1P to HDL. (**A**) Representative Western blot of ApoM expression in total plasma from chow-fed mice (*n =* 4/group). (**B**) Total S1P levels in plasma from chow-fed mice. Differences were evaluated by Student’s *t* tests. (**C**) Distribution of S1P in plasma fractionated by size-exclusion chromatography. Cholesterol levels in the control mice are shown as a reference to demonstrate the fractions in which HDL particles were eluted (gray shaded area). VLDL and LDL peaks are not shown, but were eluted in fractions approximately 25 and 35, respectively. (**D**) Distribution of S1P in lipoproteins fractions from mice fed a WTD for 3 weeks. Cholesterol levels from the control mice are shown as a reference to demonstrate the fractions in which HDL particles were eluted (gray shaded area). VLDL and LDL peaks are not shown, but were eluted in fractions approximately 21 and 31, respectively. (**E**) Western blot of ApoA1 and ApoM in lipoprotein fractions from chow diet–fed L-FoxO1,3,4 mice. (**F**) Western blot of ApoA1 and ApoM in lipoprotein fractions from Western diet–fed L-FoxO1,3,4 mice. (**G**) Plasma S1P distribution on ultracentrifuge-fractionated lipoproteins from control, CETP, L-FoxO1,3,4, and L-FoxO1,3,4:CETP mice. (**H**) Cholesterol efflux capacity of HDL isolated from Western diet–fed L-FoxO1,3,4 mice and littermate controls. Each sample is pooled HDL from 2 mice. *P < 0.05 versus control mice, by Kruskal-Wallis 1-way ANOVA; ^#^*P <* 0.05 versus CETP mice, by Mann-Whitney *U* post hoc test. Data are presented as the mean ± SEM.

Having established that FoxOs promoted ApoM expression in hepatocytes, we next examined whether liver FoxOs regulate the levels of ApoM in total plasma. Western blotting using total plasma showed that ApoM protein levels were nearly absent in male and female L-FoxO1,3,4 mice (Figure 5A and Supplemental Figure 4A). Because our genetic knockout was specific to hepatocytes, these data suggest that the majority of plasma ApoM expression arose from hepatic secretion.

By LC-MS, we found that there were no differences in total plasma S1P levels between genotypes (Figure 5B), suggesting that there were no defects in the generation or secretion of S1P into plasma. Next, we examined whether ApoM and S1P are affected in size-fractionated plasma lipoproteins from L-FoxO1,3,4 mice. Whereas control mice had 2 peaks of S1P — 1 in the HDL and 1 in the LPD fractions, presumably bound to albumin — L-FoxO1,3,4 mice showed a marked reduction of S1P in HDL and an increase in S1P in the LPD fraction (Figure 5C). (We noted that the peak of S1P in LPD was slightly shifted to the right in L-FoxO1,3,4 mice, and we speculate that albumin, which is highly sensitive to glycation [ref. 55], may elute later because of reduced glycation in L-FoxO1,3,4 mice, which have reduced hepatic glucose production [ref. 25]). Western blots of these fractions showed that ApoA1, the main apolipoprotein of HDL, was present in both genotypes. However, ApoM was nearly absent from L-FoxO1,3,4 mice (Figure 5E). Therefore, hepatic FoxOs were required for ApoM and S1P association with HDL.

We carried out the same analysis in mice that were fed the Western diet for 3 weeks. Again, we observed that in the absence of hepatic FoxOs, the levels of S1P and ApoM in HDL were largely depleted, whereas a compensatory increase of S1P in the LPD fraction was observed (Figure 5, D and F).

It has been suggested that the preferential distribution of S1P onto HDL — rather than VLDL or LDL — is regulated by cholesteryl ester transfer protein (CETP), based on experiments in WT mice transduced with an adenovirus expressing CETP at high levels (56). We used L-FoxO1,3,4 mice crossed with the CETP-transgenic mice, which express CETP at a level at which the activity is similar to that seen in human plasma (57, 58). We observed that hepatic FoxO deletion in mice on the CETP-transgenic background still had decreased HDL-S1P compared with CETP-transgenic littermate controls (Figure 5G). On the other hand, we found no differences between CETP-transgenic mice and WT controls (Figure 5G). This suggests that at physiologic levels, CETP did not have a strong effect on the lipoprotein distribution of S1P, whereas FoxOs had a strong effect on S1P distribution, even in this humanized mouse model.

The ability of HDL particles to act as acceptors of cholesterol efflux is considered an important antiatherogenic role of HDL. Thus, it was possible that the substantial decreases in ApoM and S1P on HDL in L-FoxO1,3,4 mice would affect the cholesterol efflux capacity of those particles. However, we observed no differences in the cholesterol efflux capacity between HDL isolated from control or L-FoxO1,3,4 mice (Figure 5H).

### Rescuing the expression of ApoM in L-FoxO1,3,4 mice normalizes S1P distribution.

**Figure 6:**
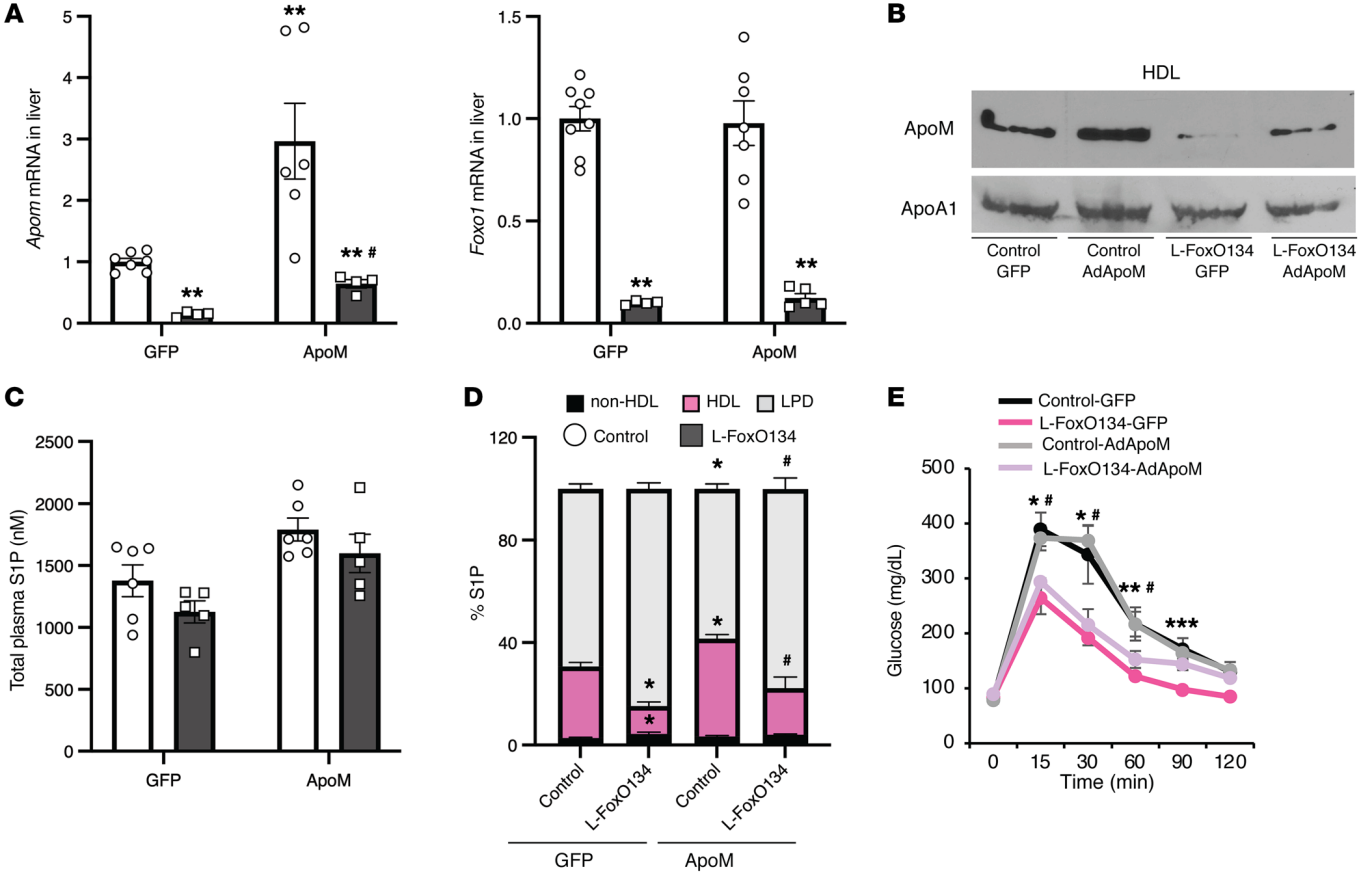
Rescuing the expression of ApoM in L-FoxO1,3,4 mice normalizes S1P distribution. Chow-fed mice were injected intravenously with murine ApoM adenovirus (0.5 × 10^9^ virus particles/gram of body weight), 8 days prior to euthanasia. (**A**) *Apom* and *Foxo1* gene expression in liver (*n =* 4–8/group). **P < 0.01 versus controls; ^#^*P <* 0.05 versus L-FoxO1,3,4-GFP. (**B**) Western blot of expression of ApoM and ApoA1 from HDL fractionated by ultracentrifugation. (**C**) Total plasma S1P levels (*n =* 5–6/group). (**D**) Plasma S1P distribution on ultracentrifuge-fractionated lipoproteins (*n =* 3–8/group). *P < 0.05 versus control-GFP; ^#^*P <* 0.05 versus L-FoxO1,3,4-GFP. (**E**) Intraperitoneal glucose tolerance test, 3 days prior to euthanasia (*n =* 4–6/group). *P < 0.05, **P < 0.01, and ***P < 0.001, control-GFP versus L-FoxO1,3,4. ^#^*P <* 0.05, control-AdApoM versus L-FoxO1,3,4-AdApoM. Statistical significance was determined by Kruskal-Wallis 1-way ANOVA with the Mann-Whitney *U* post hoc test (**A** and **C**–**E**). Data are presented as the mean ± SEM.

We next tested whether ApoM rescue in the livers of FoxO-deficient mice is sufficient to normalize S1P distribution. We transduced L-FoxO1,3,4 and control mice with low titers of an adenovirus expressing ApoM (Ad-ApoM) or a control virus (Ad-GFP). Eight days after virus injection, we harvested tissues and found that at our dose of the virus, the L-FoxO1,3,4+Ad-ApoM mice expressed levels of hepatic *Apom* mRNA similar to those in control mice transduced with Ad-GFP (Figure 6A), demonstrating efficient rescue of *Apom*. We fractionated lipoproteins by sequential density ultracentrifugation, and by Western blotting of the HDL fractions, we confirmed the rescue of ApoM protein levels in the L-FoxO1,3,4 mice (Figure 6B). Although there was no significant effect on total plasma S1P levels (Figure 6C), rescuing ApoM in L-FoxO1,3,4 mice caused a normalization of the S1P distribution (Figure 6D). The levels of other sphingolipids measured are reported in Supplemental Tables 7 and 8. These data support the hypothesis that loss of ApoM is the cause of the impaired S1P association with HDL in L-FoxO1,3,4 mice.

### Physiologic ApoM modulation does not impact glucose metabolism or triglyceride or cholesterol levels in plasma or liver.

Kurano et al. (59) overexpressed ApoM by several-fold in WT mice using adenovirus gene transfer and observed that ApoM overexpression increased glucose tolerance, potentially because of increased insulin secretion. Because FoxOs are known to regulate glucose homeostasis, we tested whether rescuing ApoM in L-FoxO1,3,4 mice affects glucose tolerance. As expected, we found that the L-FoxO1,3,4 mice had better glucose tolerance than did littermate control mice (25, 29), but rescuing ApoM in these mice had no effect (Figure 6E). Moreover, the reduced insulin levels of L-FoxO1,3,4 mice did not change by rescuing ApoM (Supplemental Figure 5A). There were no differences in body weight, total plasma triglycerides or liver cholesterol between the groups (Supplemental Figure 5, B, D, and E). As expected, L-FoxO1,3,4 mice had increased plasma total cholesterol levels (34) and liver triglycerides (25, 30), but rescuing ApoM in these mice had no effect on these phenotypes (Supplemental Figure 5, C and F). L-FoxO1,3,4 mice showed no changes in insulin tolerance (Supplemental Figure 5G). We therefore concluded that ApoM was not involved in the effects of FoxOs on glucose, triglyceride, or cholesterol homeostasis.

We also rescued ApoM in db/db mice. In db/db mice transduced with ApoM (Supplemental Figure 6A), we observed no effects on body weight, glucose tolerance, or insulin levels (Supplemental Figure 6, B–D). There were also no differences in plasma cholesterol or triglyceride levels (Supplemental Figure 6, E and F), or in hepatic cholesterol or triglyceride levels (Supplemental Figure 6, G and H). We thus concluded that ApoM was not involved in glucose, triglyceride, or cholesterol homeostasis in db/db mice.

### Reduced ApoM-S1P in L-FoxO1,3,4 mice does not affect alveolar permeability or circulating leukocytes.

*Apom^–/–^* mice have a defect in endothelial barrier function, as evidenced by excess dye permeability from blood circulation into lung tissue (4). We tested lung endothelial permeability in L-FoxO1,3,4 mice by injecting mice intravenously with Evans blue dye and measuring the ratio of dye appearance in bronchoalveolar lavage fluid (BALF) versus plasma (60). We observed no differences between the groups (Supplemental Figure 7).

**Figure 7:**
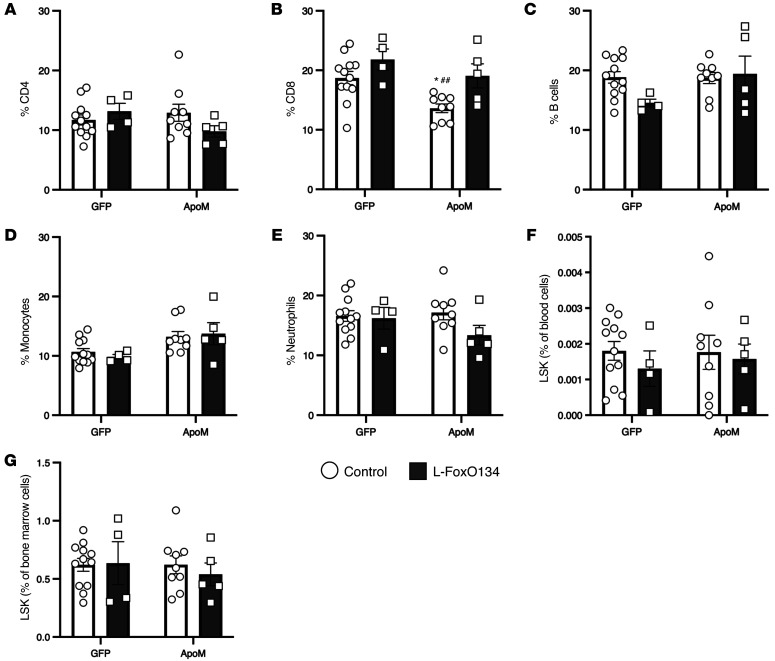
Reduced ApoM-S1P in L-FoxO1,3,4 mice does not affect circulating leukocytes. Percentage of (**A**) CD4^+^ T cells, (**B**) CD8^+^ T cells, (**C**) B cells, (**D**) monocytes, (**E**) neutrophils. (**F**) Percentage of LSK cells in blood. (**G**) Percentage of LSK cells in BM. Cells were derived from control-GFP, L-FoxO1,3,4-GFP, control-AdApoM, and L-FoxO1,3,4-AdApoM mice. *n =* 4–12/mice group for all panels. *P < 0.05 versus control-GFP; ^##^*P <* 0.01 versus L-FoxO1,3,4-GFP. Statistical significance was determined by 1-way ANOVA with Bonferroni’s multiple-comparison post hoc test. Data are presented as the mean ± SEM.

*Apom^–/–^* mice have increased circulating T and B lymphocytes as well as Lin^–^Sca^+^cKit^+^ (LSK) hematopoietic stem cells (8). We evaluated whether differences existed in the proportions of circulating leukocyte populations in L-FoxO1,3,4 mice with and without rescue of ApoM. Flow cytometric analysis revealed no reproducible differences in circulating T cells, B cells, monocytes, or neutrophils between control and L-FoxO1,3,4 mice (Figure 7, A–E, and Supplemental Figure 8, A–E). We also studied the hematopoietic stem cell population in the blood and bone marrow (BM) of L-FoxO1,3,4 mice. The percentages of LSK cells were similar between the groups (Figure 7, F and G, and Supplemental Figure 8, F and G). These results suggest that the 95% reduction of ApoM in L-FoxO1,3,4 mice had no effect on lymphopoiesis or endothelial barrier function.

Finally, we investigated whether there was any effect of the FoxO/ApoM pathway on inflammation. In liver tissue, we observed no differences in expression of *Mcp1*, *F4/80*, or *Il6* (Supplemental Figure 9, A–C). *Tnfa* gene expression showed a small increase in L-FoxO1,3,4 livers that was reversed after ApoM transduction (Supplemental Figure 9D). We also measured circulating inflammatory and antiinflammatory cytokines and found no differences between the groups (Supplemental Figure 9, E–H).

## Discussion

Our findings show that insulin-resistant humans and mice have reduced HDL-S1P levels. FoxOs promoted hepatic ApoM expression, and the FoxO/ApoM pathway was required for S1P to associate with HDL. Moreover, our data indicate that the majority of plasma ApoM arose from hepatic secretion and that hepatic ApoM was required only for HDL-associated S1P, not total plasma S1P. We recently demonstrated that hepatic FoxOs also promote clearance of HDL cholesterol by inducing the expression of *Scarb1*, encoding scavenger receptor BI (SR-BI), and *Lipc*, encoding hepatic lipase (34). Together with the data in this manuscript, these findings suggest that there are at least 2 independent mechanisms by which hepatic FoxOs regulate HDL composition.

It has been reported that individuals with type 2 diabetes have low total plasma ApoM and S1P levels (14–16) and that total plasma ApoM and S1P levels are inversely correlated with mortality in patients with diabetes (17). Moreover, low plasma ApoM levels are correlated with a risk of death in human heart failure (61). We found that HDL-S1P was lower in insulin-resistant, nondiabetic individuals in 2 independent cohorts, whereas total plasma S1P levels were unaffected. This altered distribution was directly correlated to insulin sensitivity, as assessed by hyperinsulinemic-euglycemic clamps, but not BMI or sex. Despite the preclinical evidence that HDL-ApoM-S1P has endothelium-protective effects (4, 13, 35, 36), HDL-ApoM-S1P has not been associated with endothelial function in humans. Here, we found no correlation between S1P HDL and FMD in humans, although it remains possible that the correlation could be suppressed by other factors.

FoxOs are inactivated by insulin, and it is widely believed that they are constitutively active in settings of insulin resistance (38). Interestingly, in insulin-resistant db/db and gold thioglucose–injured mice, in which FoxOs may be expected to be constitutively active, ApoM was reduced. Xu et al. previously showed that hepatic *Apom* expression and plasma ApoM levels are significantly lower in db/db mice (18). We confirmed these findings in multiple cohorts of db/db mice, at different ages (12–13 weeks and 25 weeks) and extended them by showing that db/db mice had reductions in the levels of S1P bound to HDL, with no differences in total plasma S1P levels. We also showed that this occurred in a second model of hypothalamus-centered obesity, i.e., gold thioglucose injury. Consistent with the low ApoM expression, we also found that expression levels of other FoxO targets involved in HDL homeostasis, *Scarb1* and *Lipc* (34), were also reduced in mice with hypothalamic injury. This finding is in contrast to other canonical FoxO targets in the glucose metabolism pathway, whose expression is varyingly suggestive of FoxO being active, inactive, or unaffected in these mice with hypothalamic obesity. Thus, the molecular basis underlying the decrease specifically in lipoprotein-related FoxO targets remains to be elucidated. The diet-induced obese mice had increased total plasma S1P levels, as a previous report showed (14), although S1P was not differentially distributed between the lipoproteins. Potential explanations for the differences between mice with hypothalamic obesity and diet-induced obesity may be that (a) neural inputs to liver — or adipose, which could secondarily affect liver — caused a subset of FoxOs to be inactivated, leading to decreased expression of *Apom* and potentially other FoxO targets, or that (b) the lipid content of the lard-rich obesogenic diet may have caused independent effects on S1P metabolism that overrode any effects of hepatic insulin signaling.

Our data showing that FoxO1 was sufficient to induce *Apom* in primary hepatocytes and that FoxO1 bound to the promoter and enhancer regions of *Apom* support the conclusion that *Apom* is a direct target of FoxOs’ transcriptional activity. On the other hand, we noted that after transducing mice with an adenovirus, whereby *Apom* was driven by the FoxO-independent TBP promoter, the increase in *Apom* mRNA expression was blunted in L-FoxO1,3,4 mice compared with controls (see Figure 6A). This suggests the possibility that FoxOs regulate *Apom* mRNA expression by dual mechanisms: transcriptional and posttranscriptional, perhaps through the regulation of miRNAs (62).

S1P is a signaling molecule, and it is been reported that S1P can induce differential effects, depending on its chaperone — either ApoM or albumin (4, 8, 9, 35, 36). The molecular mechanisms for the differential effects between ApoM- and albumin-bound S1P remain under investigation. It is possible that the accessibility of S1P to its receptors is different when it is bound to different chaperones. Within the structure of ApoM, S1P interacts specifically with an amphiphilic pocket in the lipocalin fold (4). Albumin serves as a promiscuous binding protein for various hydrophobic molecules, but it is not known specifically how S1P binds to albumin. This raises the possibility that being bound to ApoM allows S1P to interact more efficiently with S1P receptors, although the binding of ApoM and S1P is strong, so the spontaneous release of S1P from ApoM is unlikely (63). Another possibility invokes the preferential binding of HDL to certain cell types. SR-BI is a cell-surface receptor that binds HDL, and it has been shown to interact with S1P receptors and allow activation by HDL-bound S1P (64). Thus, S1P signaling may be considered chaperone dependent.

What is the consequence of FoxO induction of ApoM and HDL-associated S1P? One possibility we considered was that this would play a role in FoxOs’ regulation of glucose homeostasis. This was suggested by data showing that several-fold overexpression of ApoM in WT mice improves glucose tolerance and that ApoM-containing lipoproteins can stimulate insulin secretion from a mouse insulinoma cell line (59). Although L-FoxO1,3,4 mice per se have improved glucose tolerance, the rescue of ApoM in our experiment had no effect on glucose tolerance. On the other hand, db/db mice are hyperglycemic, hyperinsulinemic, and glucose intolerant, but the rescue of ApoM had no effect on these features. Previous reports have shown that the S1P metabolic pathway plays a role in triglyceride and cholesterol metabolism. Deficiency of S1P lyase increases total plasma S1P levels as well as total plasma and hepatic cholesterol and triglycerides levels (65). On the other hand, sphingosine kinase 2 inhibition reduces the levels of total plasma S1P and plasma triglycerides in LDLR^–/–^ mice (66). Furthermore, whole-body female *Apom^–/–^* mice have reduced plasma cholesterol and triglycerides (67) and increased postprandial clearance of plasma triglycerides (9). Although L-FoxO1,3,4 mice have increased plasma cholesterol and hepatic triglyceride levels, the rescue of ApoM had no effect on these phenotypes. In addition, the high cholesterol and triglyceride levels in the blood and plasma of db/db mice were unaffected by the rescue of ApoM.

We also considered the possibility that decreased hepatic expression of ApoM may have other local and systemic consequences. *Apom^–/–^* mice have an increase in lymphocytes and LSK cells in the blood and BM (8). However, the lack of ApoM in L-FoxO1,3,4 mice had no effect on immune cells or their progenitors. It also had no effect on liver or systemic inflammation or the cholesterol efflux capacity of HDL.

Several publications have suggested a role for ApoM-S1P in endothelial function, including increased phosphorylation of eNOS, decreased expression of immune cell adhesion molecules, and increased endothelial barrier function (4, 13, 35, 36). While whole-body *Apom^–/–^* mice have these defects, the low ApoM-S1P expression in L-FoxO1,3,4 mice did not impair endothelial function.

How can these findings be interpreted in light of the data from *Apom^–/–^* mice? L-FoxO1,3,4 mice had a near-total absence (~90% reduction) of hepatic ApoM mRNA and protein expression and a similarly profound reduction in plasma ApoM. This reduction in ApoM caused a substantial reduction in HDL-associated S1P. However, there was a compensatory increase in S1P bound to albumin, such that L-FoxO1,3,4 mice had no differences in total plasma S1P levels compared with levels in the control mice. These findings indicate that (a) most ApoM in plasma arises from hepatocytes and (b) hepatic ApoM is either not required to maintain total plasma S1P content (i.e., albumin-S1P content), or very low levels of ApoM are sufficient. In contrast, whole-body *Apom^–/–^* mice have a reduction in total plasma S1P of 46% compared with levels in WT mice (4). The contrast between these 2 mouse models suggests the possibilities that (a) very minute amounts of ApoM are sufficient to carry out its effects on endothelial function and lymphopoiesis, and/or (b) some of the phenotypes of *Apom^–/–^* mice are attributable to a deficiency in total plasma S1P, and/or (c) nonhepatocyte cells that express ApoM may be involved in maintaining total plasma S1P levels.

Overall, these findings support a link between hepatic insulin signaling and HDL composition through FoxO transcription factors. Our human and mouse data suggest the possibility that the endothelial and lymphopoietic defects observed in the total-body *Apom^–/–^* mice may not occur in response to a partial reduction of HDL-S1P in humans or mice. Emerging evidence points to potentially important roles of HDL-ApoM-S1P in heart failure and kidney disease (61, 68), and it will be of interest to examine whether this complex links insulin action to those pathologies. Further studies on the effects of ApoM-S1P will be of great relevance to understanding this evolutionarily conserved complex and its effects on insulin resistance and its vascular complications.

## Methods

### Study participants.

Cohort 1 included 40 participants. The participants fasted overnight prior to plasma collection. Hyperinsulinemic-euglycemic clamps were carried out using Humulin R (Lilly) infused at a rate of 240 pmol/min/m^2^ for 120 minutes as described previously (37). These 40 individuals were a subset of a larger cohort composed of 143 nondiabetic volunteers (*n* = 50 women, *n* = 93 men), aged 18–56 years, with a BMI between 18.8 and 48.0 kg/m^2^; the 20 individuals with the lowest M value (the rate of glucose disposal) and the 20 individuals with the highest M value on the hyperinsulinemic-euglycemic clamp were chosen to represent the extremes of insulin sensitivity distribution for analysis in the present study.

Cohort 2 included 81 participants. Study eligibility included participants who (a) were aged 18–55 years; (b) had no nicotine use in the past year, no history of substance or alcohol dependency in the 10 years prior to study entry, and a negative urine toxicology screen; (c) were taking no prescribed cardiovascular, carbohydrate, endocrine, or psychiatric medications; (d) had no history of diagnosed cardiovascular, metabolic, or endocrine disorders; and (e) for women, were not pregnant and had regular menstrual cycling (26–35 days) for the 3 months before study entry. The participants fasted overnight prior to plasma collection. Hyperinsulinemic-euglycemic clamps were carried out using Humulin R (Lilly), infused at a rate of 240 pmol/min/m^2^ for 150 minutes, as described previously (37). The participants were classified according their rate of insulin-mediated glucose disposal; 39 participants were classified as insulin sensitive (M >4.5 mg/min/kg), and 42 participants were classified as insulin resistant (M ≤4.5 mg/min/kg). Endothelium-dependent FMD via brachial artery–reactive hyperemia testing was performed as previously described (37).

### Mice and diets.

All mice were maintained on a 12-hour light/12-hour dark cycle, with the dark cycle occurring between 7:00 pm and 7:00 am. L-FoxO1,3,4 mice have been previously described (25, 69). Mice that were 12 weeks or older and 2-day-old mice of both sexes were used in the studies. For the acute FoxO depletion experiments, mice were fed a standard chow diet (Purina). Adult male mice bearing *Foxo1^fl/fl^*, *Foxo3^fl/fl^*, and *Foxo4^fl/Y^* alleles were transduced with an AAV (serotype 8) expressing Cre recombinase driven by the hepatocyte-specific Tbg promoter (AAV8.Tbg.Cre) or with control virus (AAV.GFP). Mice were injected intravenously with 1 × 10^11^ virus particles/mouse, 4 weeks prior to euthanasia. AAV8.Tbg.Cre was a gift of Morris Birnbaum (Perelman School of Medicine, University of Pennsylvania, Philadelphia, Pennsylvania, USA). For the adenovirus experiments, adult male mice were injected intravenously with murine ApoM adenovirus (Welgen) at 0.5 × 10^9^ virus particles per gram of body weight, 8 days prior to euthanasia, and were fed a standard chow diet. CETP mice have been previously described (57, 58). L-FoxO1,3,4 mice were crossed with CETP-transgenic mice and fed a standard chow diet. For the studies of ApoM and S1P distribution, control and L-FoxO1,3,4 mice were fed either a standard chow diet (Purina) or a Western-type diet (WTD), containing 42% kcal from fat and 0.2% cholesterol (Harlan Teklad, TD.88137). For the db/db studies, male db/db and db/+ mice were purchased from The Jackson Laboratory and were studied when they were 12, 13, or 25 weeks old. For the diet-induced obesity studies, male C57BL/6J mice were purchased from The Jackson Laboratory when they were 6 weeks old, and they were fed either a standard chow diet (Purina) or a HFD containing 60% kcal from fat (Research Diets, D12492) until they were 13 or 29 weeks old. For gold thioglucose experiments, 14- to 27-week-old male control and L-FoxO1,3,4 mice were treated with 0.6 g/kg gold thioglucose or an equivalent volume of saline and continued to receive a chow diet for 13 weeks. Five control mice that were injected with gold thioglucose remained lean, presumably due to ineffective gold thioglucose injection, and were thus excluded from analysis. Two L-FoxO1,3,4 mice that were injected with gold thioglucose also remained lean, and these were analyzed together with the other nonobese (saline-injected) L-FoxO1,3,4 mice, because we had only small numbers of knockout mice.

### Primary hepatocytes studies.

Primary hepatocytes were isolated from male mice via collagenase perfusion, as previously described (32). Cells were plated on collagen-coated cultureware for 2 hours. Following attachment, hepatocytes were transduced with FoxO1-ADA (T24A, S253D, and S316A mutations; ref. 45), FoxO1-ADA-DBD T24A, S253D, and S316A mutations (ADA) plus N208A and H212R mutations (DBD) (46), or FoxO1-Δ256 (AA1-256, truncated form; ref. 52) for 16 hours.

### mRNA and protein expression.

Liver and hepatocyte RNA was extracted using TRIzol (Invitrogen, Thermo Fisher Scientific). cDNA was generated using the High-Capacity cDNA Reverse Transcription Kit (Applied Biosystems). qPCR was performed with iTaq Universal SYBR Green Supermix (Bio-Rad). *36b4* was used as housekeeping gene for normalization. The primer sequences are listed in Supplemental Table 9.

For Western blotting, primary antibodies directed against the following proteins were used: ApoM (LSBio, catalog LS-C319551), ApoA1 (Meridian Life Science, catalog K23500R), and β-actin (Cell Signaling Technology, catalog 4970).

### ELISA cytokine measurement.

Plasma IL-1b, IL-6, IL-10, and TNF-α levels were measured using the MILLIPLEX MAP Mouse Cytokine/Chemokine Magnetic Bead Panel (MilliporeSigma, MCYTOMAG-70K). Standard curves were generated using the specifics standards supplied by the manufacturer. Samples were assayed according to the manufacturer’s instructions using a Luminex 200 system (Luminex).

### ChIP.

Male FoxO1-Venus mice were fed either chow or a HFD (60% of kcal from fat, D12492i, Research Diets) for 4 weeks, starting at 8 weeks of age. ChIP of liver tissue was performed as previously described (53). The primer sequences used are listed in Supplemental Table 9.

### Metabolic tests.

Blood glucose was measured using the Breeze2 monitor and strips (Bayer). Insulin ELISAs were from MilliporeSigma. For intraperitoneal glucose tolerance tests, mice were fasted for 16 hours and injected intraperitoneally with glucose (2 g/kg). We obtained blood samples 0, 15, 30, 60, 90, and 120 minutes after the injection and measured glucose levels. For the intraperitoneal insulin tolerance tests, the mice were fasted for 5 hours and injected intraperitoneally with 0.75 U insulin (Humalog, Lilly). Total cholesterol and triglyceride levels were measured using a colorimetric assay from Wako. Liver lipids were extracted in chloroform/methanol, as described previously (70).

### Plasma lipoprotein analysis.

Non-HDL (d <1.063 g/mL), HDL (1.063 <d <1.210 g/mL), and LPD (d >1.210 g/mL) fractions were separated by sequential density ultracentrifugation using NaBr buffers. Total plasma (70 μL) was separated using the Optima MAX-TL Ultracentrifuge with the TLA-100 rotor (Beckman Coulter). Plasma lipoproteins were also analyzed by running 200 μL plasma onto a fast protein LC system consisting of a Superose 6 10/300 GL column (Amersham Pharmacia Biotech), and fractions from chow-fed mice were collected using the fraction collector FC-204 (Gilson), whereas fractions from Western diet–fed mice were collected using the fraction collector FRAC-100 (Pharmacia LKB).

### Quantification of S1P, sphingosine, SP1, and sphinganine.

Bioactive sphingolipids were measured in total plasma or non-HDL, HDL, and LPD fractions using ultra-performance liquid chromatography–tandem mass spectrometry (UPLC-MS/MS) and quantitated using stable isotope–labeled internal standards. Samples were spiked with 20 μL of a 2 μM internal standard mixture (C17 sphingosine, C17-sphinganine, C17-1- P sphingosine and C17-1-P sphinganine; Avanti Polar Lipids) and extracted by mixing with 300 μL methanol. The mixture was vortexed well and centrifuged at 3000*g* for 10 minutes at 4°C. The clear upper phase was evaporated under nitrogen, and the extracted lipids were reconstituted in 70 μL methanol and transferred into LC-MS vials for analysis. LCMS analysis was performed using a Waters Xevo TQ MS ACQUITY UPLC system. Five microliters of the sample were loaded onto a Waters ACQUITY UPLC BEH Phenyl column (3 mm × 100 mm, 1.7 μm) maintained at 40°C. The UPLC flow rate was 300 μL/min with the following mobile phases: solvent A (H_2_O, containing 0.2% formic acid and 1 mM ammonium formate) and solvent B (methanol, containing 0.2% formic acid and 1 mM ammonium formate). Solvent B was maintained at 50% for 2 minutes and increased to 95% for 0.1 minute, held for the subsequent 4.5 minutes, and then brought back to initial conditions and reconditioned for 1.5 minutes. Positive electrospray ionization MS/MS (ESI-MS/MS) under multiple reaction monitoring (MRM) mode was performed using the following parameters: capillary voltage, 4 kV; source temperature, 150°C; desolvation temperature, 500 °C; desolvation gas flow, 1000 L/h; and collision energy, 18 eV. The MRM transitions were as follows: C18 sphingosine 300.3 → 252.3; C18 sphingosine-1-P 380.3>264.3; C18 sphinganine 302.3 → 254.3; C18 sphinganine-1-P 382.3>266.3, C17 sphingosine 286.3>238.2; C17 sphingosine-1-P 366.3 → 250.2; C17 sphinganine 288.3>240.2; and C17 sphinganine-1-P 368.3 → 252.2. Quantification was done at the Biomarkers Core facility of the Irving Institute for Clinical and Translational research at Columbia University.

### Alveolar permeability analysis.

A sterile solution of Evans blue dye (Fisher Chemical) in 4 g/dL albumin at a molar ratio of 1:4 in PBS was prepared the day before the experiment. Evans blue dye–albumin (200 μlL) was intravenously injected into mice 4 hours before they were euthanized. Blood was collected from the heart. BALF from both lungs was collected by instillation and aspiration of 1 mL ice-cold, Ca^2+^-free PBS via tracheal cannula (3 times). Absorbance at 625 nm was measured from plasma and BALF supernatant. Alveolar permeability was calculated as the ratio of absorbance in BALF versus plasma.

### Flow cytometric analysis of blood and BM.

For blood leukocytes, an aliquot of whole blood obtained from a cardiac puncture was placed into EDTA-coated tubes to prevent coagulation and kept on ice to prevent leukocyte activation. A 30 μL aliquot was used for automated cell counting, with remaining cells subjected to RBC lysis. A portion of WBCs were then washed and stained with an antibody cocktail containing anti-CD45, anti-CD115, anti-Gr1, anti-TCRβ, anti-CD19, anti-CD4, and anti-CD8b antibodies for 30 minutes in the dark on ice. Gated from the CD45^+^ leukocytes, monocytes were identified as CD115^+^ and included both the Ly6-C^hi^ and Ly6-C^lo^ (Gr1) subsets. Neutrophils were identified as CD115^–^ and Ly6-C^hi^. T and B lymphocytes were identified as TCRβ^+^CD19^–^ and CD19^+^TCRβ^–^, respectively, from the CD115^–^Gr1^–^ cell population, with the CD4^+^ and CD8^+^ T lymphocytes further gated from the TCRβ^+^CD19^–^ cell population. Hematopoietic stem cells (LSKs) were measured in the blood or in BM. Briefly, BM from femurs and tibias was flushed with PBS through a cell strainer (40 μm) before RBC lysis, centrifugation, and washing. BM (or the remaining WBC portion) was resuspended in a cocktail of antibodies against lineage-committed (lin) cells (B220, CD19, CD11b, CD3e, TER119, CD2, CD8b, CD4, GR1: all FITC), Sca-1, and c-Kit. LSK cells were identified as lin^–^cKit^+^Sca1^+^. All samples were run on a BD Fortessa flow cytometer and analyzed using FlowJo software (TreeStar). Antibodies were diluted 1:400, and their details are provided in Supplemental Table 10. Blood leukocyte are expressed as a percentage of CD45^+^ cells. LSKs are reported as a percentage of extracted BM or blood cells (after RBC lysis).

### Cholesterol efflux capacity.

HDL was isolated from L-FoxO1,3,4 mice and littermate controls using sequential density ultracentrifugation as described above. Isolated HDL samples from 2 mice were pooled per sample. The protein concentration in isolated, pooled HDL was measured by bicinchoninic acid (BCA) assay. BM-derived macrophages from WT C57BL/6J mice were prepared and grown in L cell media consisting of DMEM, 10% FBS, 1% penicillin/streptomycin, and 20% conditioned media from L929 cells. Seven days after isolation, the media were changed to DMEM containing 0.2% FFA-free BSA, 100 μg/mL AcLDL, 3 μM TO901317, and 2 μCi/mL ^3^H-cholesterol (PerkinElmer) to induce foam cell formation. After 24 hours, macrophages were carefully washed and then incubated for 6 hours with DMEM containing 0.2% FFA-free BSA alone, or with the addition of 50 μg human HDL or 50 μg or 10 μg pooled, isolated HDL from L-FoxO1,3,4 mice or control mice. The media were then collected. Cells were lysed in 0.1 M NaOH. ^3^H was quantified from the media and cell lysates by liquid scintillation counting. Results are expressed as media counts as a percentage of total counts.

### Statistics.

Statistical analysis was performed using SPSS 23.0 statistical software (IBM). Data are expressed as the mean ± SEM. In the human studies, data are expressed as the mean ± SEM or the median (IQR) for normally or nonnormally distributed variables, respectively. Differences between 2 groups were assessed by 2-tailed Student’s *t* test, and differences among more than 2 groups were evaluated by 1- or 2-way ANOVA, followed by Bonferroni’s post hoc test or by Kruskal-Wallis 1-way ANOVA, followed by Mann-Whitney *U* post hoc test. Multiple regression was performed by standard methods. A *P* value of 0.05 or less was considered statistically significant. Statistical parameters are shown in the figure legends.

### Study approval.

All animal protocols were approved by the IACUC of the Columbia College of Physicians and Surgeons, and the human studies were conducted under the approval of the IRB of the University of Miami. Written informed consent was obtained from each study participant.

## Author contributions

MCI and RAH conceptualized the study. MCI, MJK, MW, TK, and GAG designed the methodology. MCI, NS, SXL, MJK, MW, TK, MH, JRC, KZ, EM, IO, and NR performed experiments. RAH, SC, TGU, EF, and BEH provided resources. MCI and RAH wrote the original draft of the manuscript. All authors wrote, reviewed, and edited the manuscript. MCI, BEH, and RAH acquired funding for the study.

## Supplementary Material

Supplemental data

## Version 1 02/01/2022

In-Press Preview

## Version 2 04/01/2022

Electronic publication
