# Supplementary material for: Hepatic FoxOs link insulin signaling with plasma lipoprotein metabolism through an apolipoprotein M/sphingosine-1-phosphate pathway
Source: J Clin Invest. 2022 Apr 1;132(7):e146219. doi: 10.1172/JCI146219 (PMC8970673; doi:10.1172/JCI146219)
Supplement: Supplemental data [file jci-132-146219-s105.pdf]

Supplementary figure 1

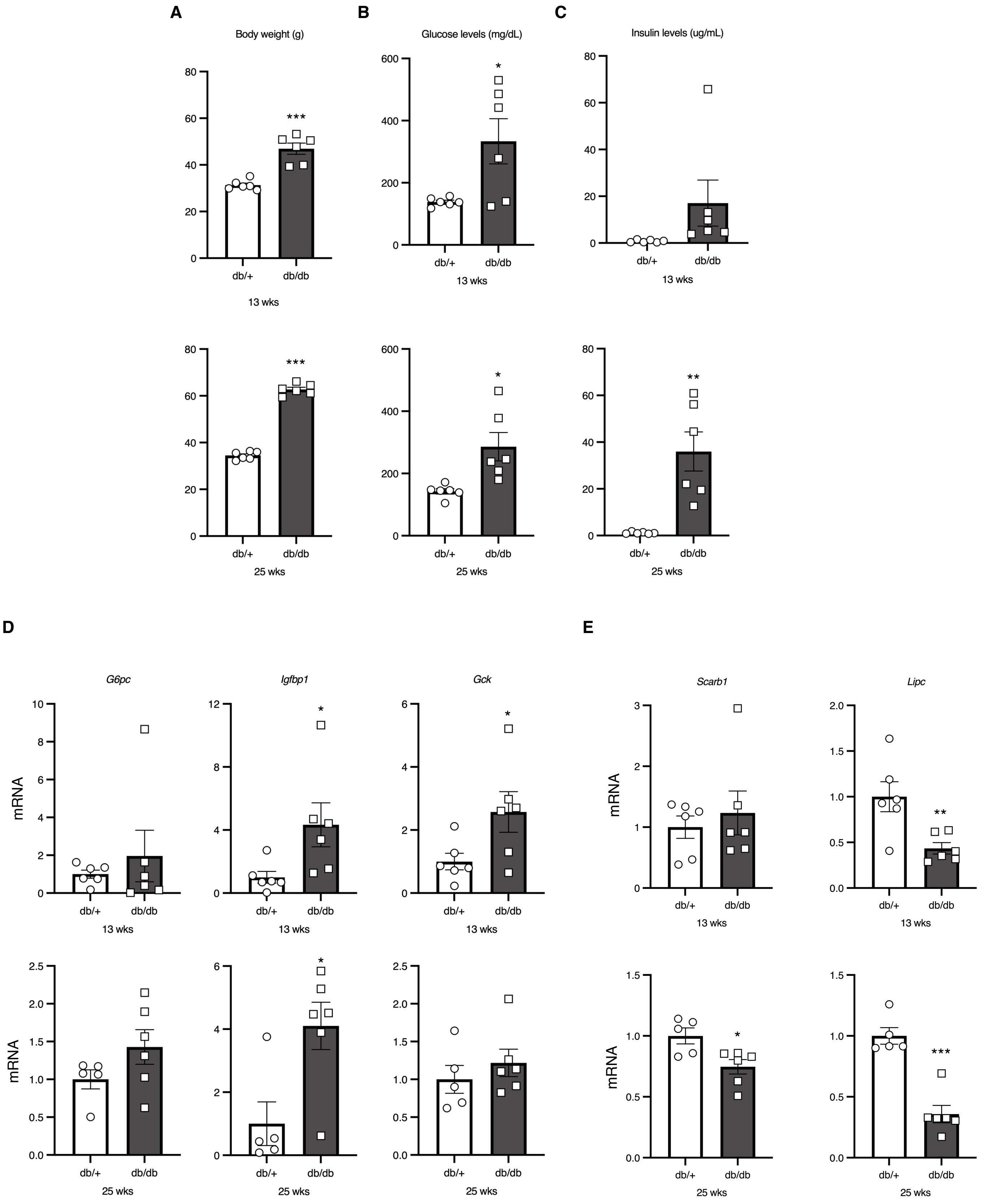

Supplementary Figure 1. Metabolic parameters and hepatic gene expression in db/db mice. Top: 13 week-old mice, Bottom: 25 week-old mice. **A)** Total body weight. **B)** Plasma glucose levels after 5 hours fasting. **C)** Plasma insulin levels after 5 hours fasting. **D)** *G6pc, Gck* and *Igfbp1* gene expression in liver **E)** *Scarb1* and *Lipc* gene expression in liver. (n=5-6/group for all panels) Data are presented as mean ± SEM. *P<0.05, **P<0.01, ***P<0.001 by student's t-tests.

Supplementary figure 2

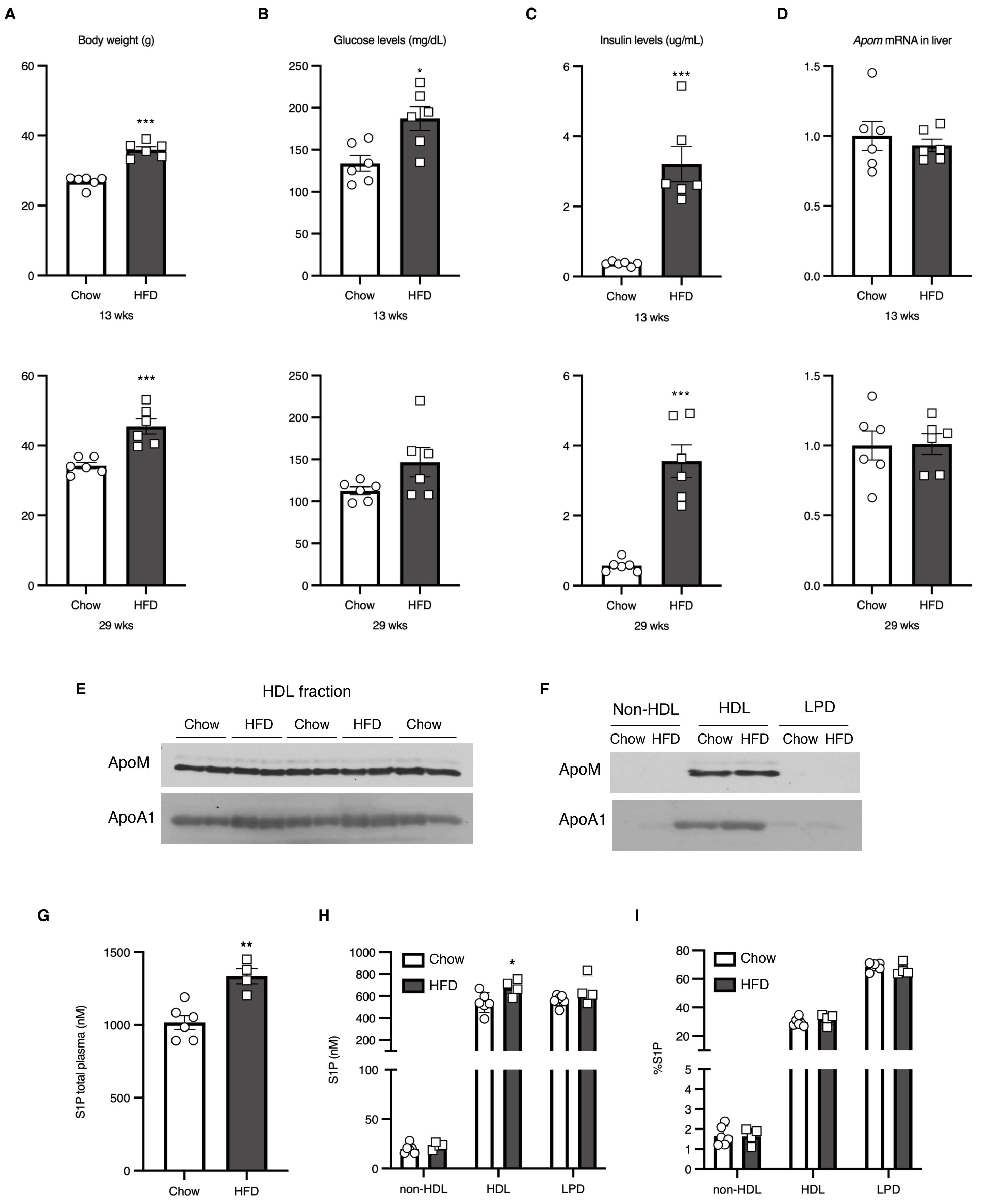

Supplementary Figure 2. Metabolic parameters, hepatic gene expression, HDL-ApoM and S1P levels in diet induced obese mice. Top: 13 week-old mice, Bottom: 29 week-old mice. HFD=high fat diet. **A)** Total body weight. **B)** Plasma glucose levels after 5 hours fasting. **C)** Plasma insulin levels after 5 hours fasting. **D)** *Apom* gene expression in liver. **E)** Western blot of ApoM and ApoA1 in HDL fractionated by sequential density ultracentrifugation from plasma of 13 week-old mice. **F)** Representative western blot of ApoM and ApoA1 in ultracentrifuge-fractionated plasma from 13 week-old mice. **G**) Total plasma S1P levels. **H**) Plasma S1P distribution. **I**) Percentage of S1P distribution. LPD=lipoprotein depleted. (n=4-6/group for all panels) Data are presented as mean± SEM. *P<0.05, **P<0.01, ***P<0.001 by student's t-tests.

Supplementary figure 3

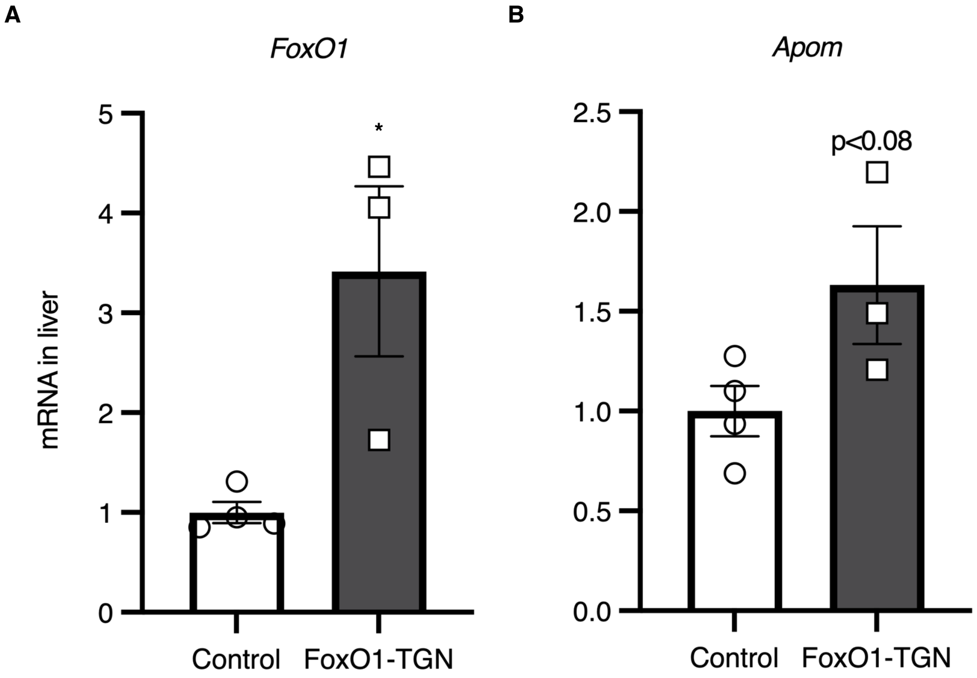

Supplementary Figure 3. Hepatic FoxO1 and ApoM expression in FoxO1 transgenic mice. A) FoxO1 and **B)** ApoM gene expression in liver. (n=3/group). Data are presented as mean ± SEM. *P<0.05, by student's t-tests.

Supplementary figure 4

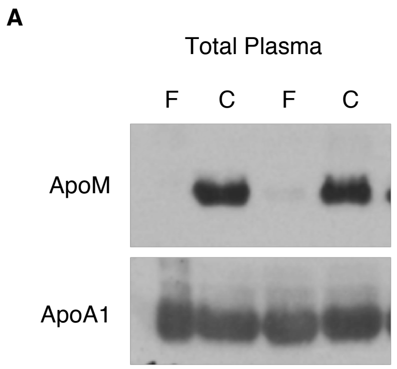

Supplementary Figure 4. Plasma ApoM expression in L-FoxO1,3,4 female mice. A) Representative western blot of ApoM and ApoA1 in total plasma from chow-fed mice. F=L-FoxO1,3,4 mice, C=littermate control mice (n=7/group).

Supplementary figure 5

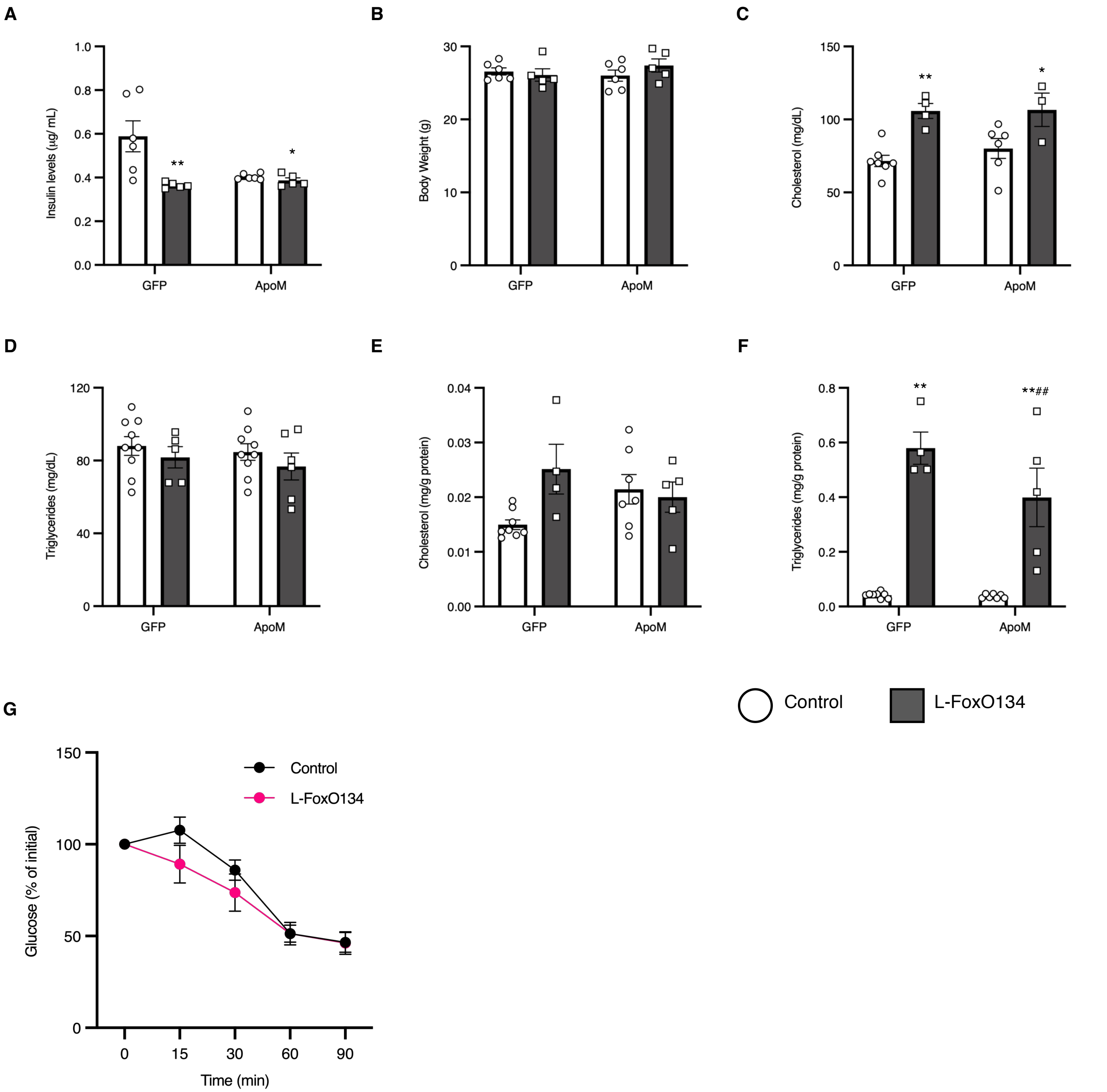

Supplementary Figure 5. Metabolic parameters and hepatic cholesterol and triglyceride levels in L-FoxO1,3,4 mice after rescue of ApoM. A) Insulin levels after 5 hours fasting (n=5-6/group). **B)** Total body weight (n=5-6/group). **C**) Total cholesterol levels in plasma after 5 hours fasting (n=3-7/group). **D**) Total triglycerides levels in plasma after 5 hours fasting (n=5-9/group). **E**) Hepatic cholesterol levels (n=4-8/group). **F)** Hepatic triglyceride levels (n=4-8/group). *P<0.05, **P<0.01 vs control-GFP mice, ##P<0.01 vs control-AdApoM by Kruskal-Wallis 1-way ANOVA and Mann-Whitney U post hoc test. **G)** Insulin tolerance tests in chow-fed L-FoxO1,3,4 mice and littermate controls (n=5-6 per group). Analysis by student's t-test. Data are presented as mean ± SEM.

Supplementary figure 6

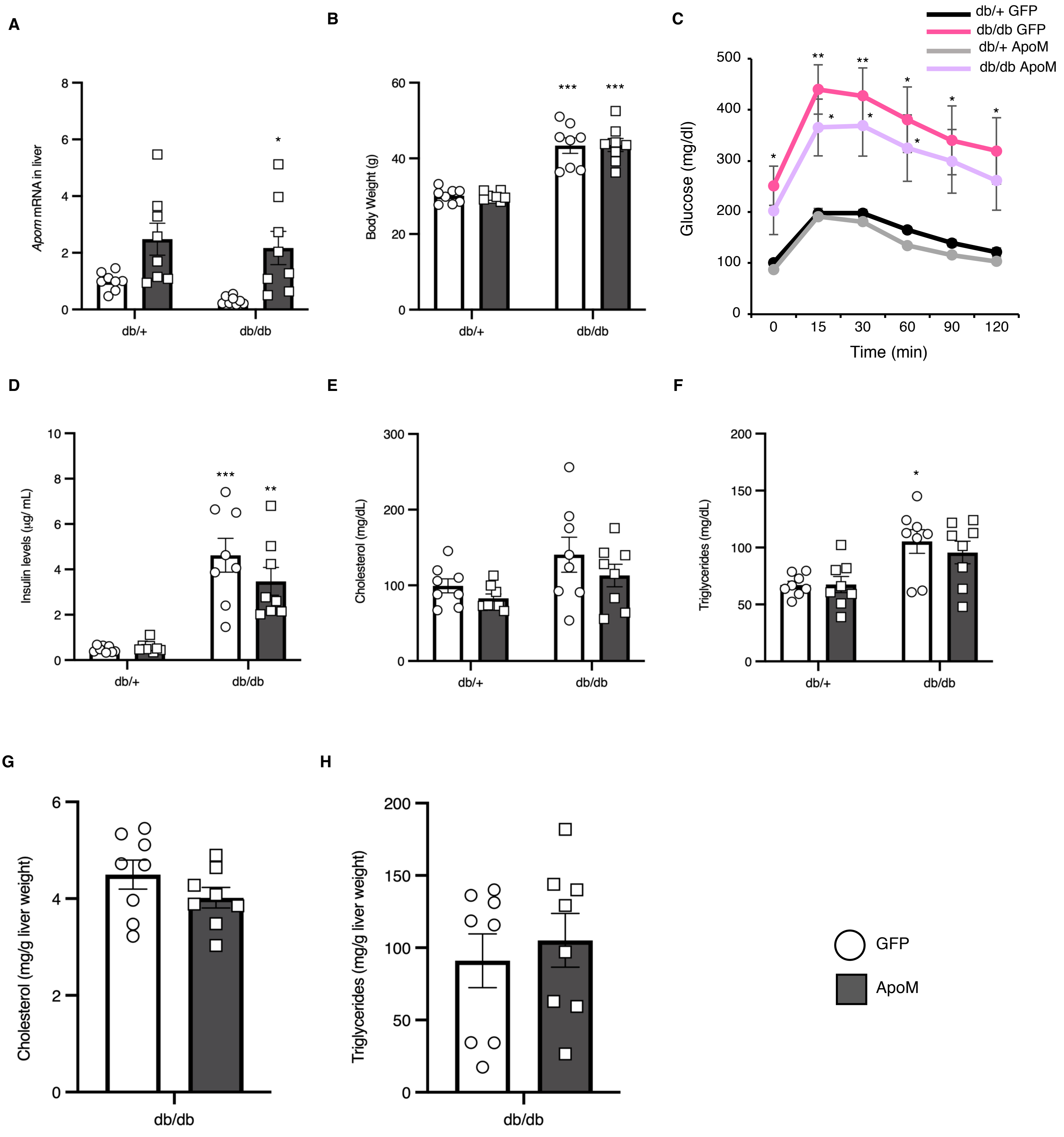

Supplementary Figure 6. Rescue of ApoM in db/db mice. **A)** *Apom* gene expression in liver. **B**) Total body weight. **C)** Intraperitoneal glucose tolerance test **D**) Insulin levels after 5 hours fasting. **E**) Total cholesterol levels in plasma. **F**) Total triglycerides levels in plasma **G)** Hepatic cholesterol levels. **H)** Hepatic triglyceride levels (n=8/group for all the panels). Data are presented as mean ± SEM. *P<0.05, **P<0.01, ***P<0.001 by 1-way ANOVA and Bonferroni multiple comparation post hoc test.

Supplementary figure 7

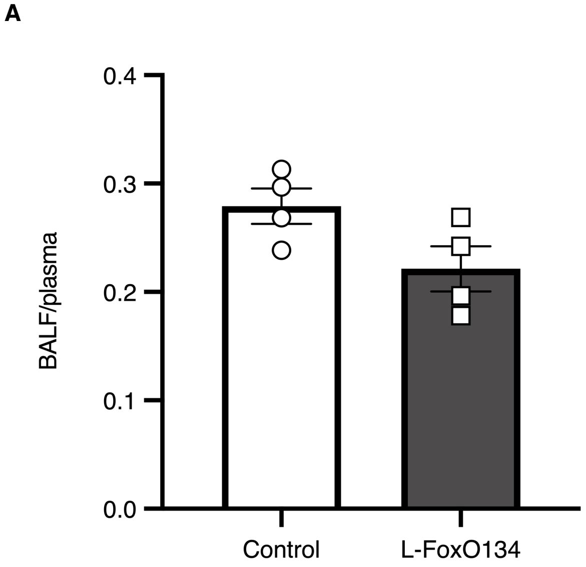

Supplementary Figure 7. **Alveolar permeability in L-FoxO1,3,4 mice**. BALF= Bronchoalveolar lavage fluid. Ratio bronchoalveolar lavage evans blue dye versus plasma evans blue dye (n=4/group). Data are presented as mean ± SEM. Analysis by student's t-tests.

Supplementary figure 8

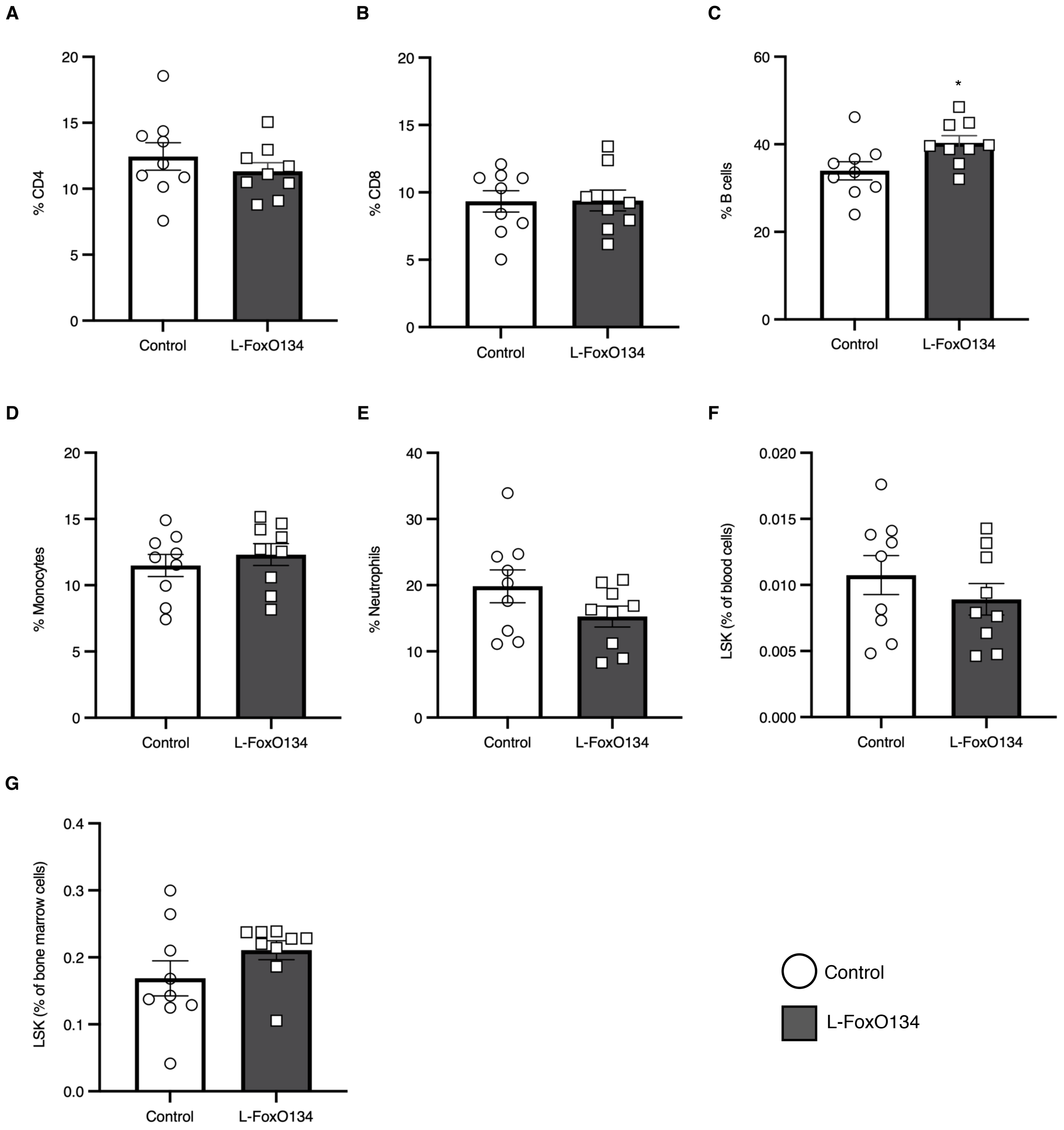

Supplementary Figure 8. **Circulating leukocytes in L-FoxO1,3,4 mice. A**) $CD4^+$ T cells. **B**) $CD8^+$ T cells. **C**) B cells. **D**) Monocytes **E**) Neutrophils. **A-E)** are percentages of blood $CD45^+$ cells. **F**) Percentage of LSK cells in blood. **G**) Bone marrow LSK cells. From Control and L-FoxO1,3,4 mice. n=8/group for all panels. *P<0.05 vs Control. Analysis by student's t-tests. Data are presented as mean ± SEM.

Supplementary figure 9

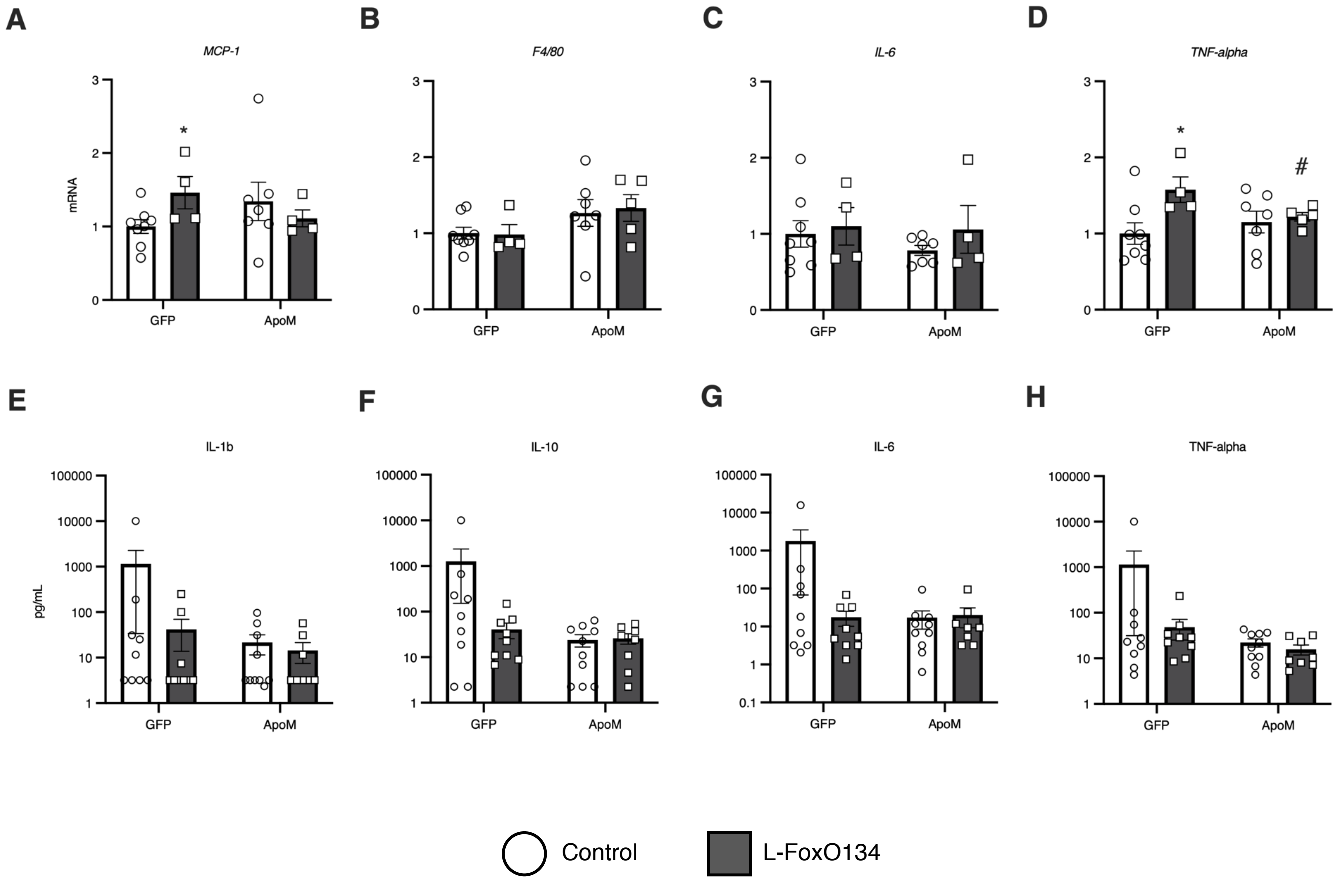

Supplementary Figure 9. Liver and systemic inflammation in L-FoxO1,3,4 mice after rescue of ApoM. **A-D**) Hepatic gene expression (n=4-8/group). **E)** IL-1b, **F)** IL-10, **G)** IL-6, **H)** TNF-alpha plasma levels from Control-GFP, L-FoxO1,3,4-GFP, control-AdApoM, L-FoxO1,3,4-AdApoM mice.(n=8-10/group). Data are presented as mean ± SEM. *P<0.05 vs control-GFP mice, #P<0.05 vs L-FoxO134-GFP mice by Kruskal-Wallis 1-way ANOVA and Mann-Whitney U post hoc test.

Supplementary Table 1. Clinical characteristics of subjects in the discovery cohort.

	Insulin Sensitive	Insulin Resistant	p-value
**N (M/F)**	20 (12/8)	20 (15/5)	-
**Age (years)**	34 ± 9	41 ± 6	0.006
**Race/Ethnicity**	Caucasians	Caucasians	–
**BMI (kg/m^2)**	24.7 ± 3.9	31.8 ± 2.5	<.0001
**FPG (mmol/L)**	4.8 ± 0.5	5.5 ± 0.6	0.0004
**FPI (pmol/L)**	58 [29]	142 [89]	<.0001
**Total cholesterol (mmol/L)**	4.6 ± 0.5	4.8 ± 0.9	ns
**HDL-C (mmol/L)**	1.5 ± 0.4	0.9 ± 0.2	<0.0001
**Triglycerides (mmol/L)**	0.88 ± 0.28	2.45 ± 1.77	0.0002
**M (µmol·min^{-1}·kg$_{lbm}$$^{-1}$)**	87 [17]	10 [5]	<.0001

Data are expressed as mean ± standard error or median [interquartile range]. BMI=body mass index, FPG=fasting plasma glucose, FPI=fasting plasma insulin, lbm=lean body mass, M=glucose disposal rate, ns=not significant.

Supplementary Table 2. Clinical characteristics of the subjects in the validation cohort.

	Insulin Sensitive	Insulin Resistant	p-value
**N (M/F)**	39 (27/12)	42 (27/15)	-
**Age (years)**	37 ± 1	41 ± 1	0.01
**Race/Ethnicity (%)**			ns
**Black**	10.3	14.3	
**Hispanic white**	76.9	78.6	
**Non-Hispanic white**	10.3	4.8	
**Other**	2.6	2.4	
**BMI (kg/m^2)**	25.5 ± 0.5	30.7 ± 0.5	<0.0001
**FPG (mmol/L)**	4.8 ± 0.07	5.1 ± 0.1	0.01
**FPI (pmol/L)**	56 [36]	87 [42]	0.001
**Total cholesterol (mmol/L)**	4.7 ± 0.1	4.9 ± 0.1	ns
**HDL-C (mmol/L)**	1.2 ± 0.04	1.0 ±0.04	0.0001
**Triglycerides (mmol/L)**	1.08 ±0.08	1.99±0.20	<0.0001
**M (µmol·min^{-1}·kg$_{lbm}$$^{-1}$)**	60 [29]	22 [17]	<0.0001

Data are expressed as mean ± standard error or median [interquartile range]. BMI=body mass index, FPG=fasting plasma glucose, FPI=fasting plasma insulin, lbm=lean body mass, M=glucose disposal rate, ns=not significant.

Supplementary Table 3. Sphingolipid levels in the human discovery cohort.

		Total Plasma			non-HDL			HDL			LPD		
		IS	IR	p-value	IS	IR	p-value	IS	IR	p-value	IS	IR	p-value
C18 Sphingosine	nM	30.32 ± 2.3	28.62 ± 1.5	ns	22.75 ± 1.3	25.74 ± 3.4	ns	18.74 ± 1.3	15.01 ± 0.8	0.02	21.52 ± 1.5	22.52 ± 1.8	ns
	%				34.09 ± 1.7	36.38 ± 2.7	ns	19.14 ± 1.5	15.19 ± 1.1	0.04	46.76 ± 2.4	48.43 ± 2.9	ns
C18 Sphinganine	nM	10.96 ± 0.7	10.99 ± 0.6	ns	9.98 ± 0.4	11.13 ± 1.3	ns	7.03 ± 0.4	6.82 ± 0.4	ns	10.42 ± 0.4	8.85 ± 0.4	0.009
	%				33.16 ± 1	37.43 ± 1.9	0.05	15.71 ± 0.9	16.48 ± 1.3	ns	51.13 ± 1.4	46.1 ± 1.3	0.01
C18 Sphinganine-1-phosphate	nM	91.71 ± 3.1	91.71 ±3.6	ns	31.21 ± 1.2	31.61 ± 2.1	ns	51.93 ± 1.6	51.73 ± 1.4	ns	83 ± 2.3	86.3 ± 2.9	ns
	%				16.59 ± 0.5	16.29 ± 0.9	ns	18.43 ± 0.5	17.95 ± 0.5	ns	65 ± 0.8	65.76 ± 1	ns

Data are expressed as mean ± standard error. LPD=Lipoprotein Depleted. IS=Insulin Sensitive, IS=Insulin Resistant, ns=not significant.

Supplementary Table 4. Sphingolipid levels in the human validation cohort.

		Total Plasma			non-HDL			HDL			LPD		
		IS	IR	p-value	IS	IR	p-value	IS	IR	p-value	IS	IR	p-value
C18 Sphingosine	nM	10.45 ± 0.9	11.85 ± 0.6	ns	5.83 ± 0.5	5.53 ± 0.3	ns	5.64 ± 0.5	5.27 ± 0.5	ns	7.84 ± 0.5	7.03 ± 0.4	ns
	%				27.59 ± 1.7	29.47 ± 1.7	ns	18.04 ± 1.4	18.12 ± 1.5	ns	54.37 ± 2.1	52.42 ± 1.9	ns
C18 Sphinganine	nM	2.51 ± 0.3	2.46 ± 0.3	ns	0.94 ± 0.1	0.80 ± 0.1	ns	0.75 ± 0.1	0.68 ± 0.1	ns	1.32 ±0.2	1.54 ± 0.2	ns
	%				30.03 ± 2.8	28.32 ± 2.9	ns	17.58 ± 2	16.13 ± 1.9	ns	52.4 ± 3.2	55.55 ± 3.3	ns
C18 Sphinganine-1-phosphate	nM	110.39 ± 6.6	111.56 ± 6.1	ns	14.96 ± 0.8	15.82 ± 0.7	ns	40.71 ± 1.9	36.57 ± 1.7	ns	65.08 ± 5	61.61 ± 4.9	ns
	%				11.31 ± 0.5	12.97 ± 0.6	0.04	20.81 ± 0.9	19.93 ± 1	ns	67.88 ± 1.1	67.1 ± 1.4	ns

Data are expressed as mean ± standard error. LPD=Lipoprotein Depleted. IS=Insulin Sensitive, IS=Insulin Resistant, ns=not significant.

Supplementary Table 5. Sphingolipid levels in the db/db mice.

		Total Plasma			VLDL+LDL			HDL			LPD		
		db/+	db/db	p-value	db/+	db/db	p-value	db/+	db/db	p-value	db/+	db/db	p-value
C18 Sphingosine	nM	38.65 ± 6	70.24 ± 13.6	ns	17.35 ± 5.6	12.89 ± 1.7	ns	33.34 ± 4.6	53.3 ± 9.6	ns	40.28 ± 4.5	64.22 ± 12.2	ns
	%				17.48 ± 5.3	10.31 ± 2.1	ns	22.46 ± 3.2	24.3 ± 2.2	ns	60.06 ± 6.1	65.4 ± 2.8	ns
C18 Sphinganine	nM	17.35 ± 1.9	32.5 ± 8.3	ns	5.45 ± 2.3	6.85 ± 2.1	ns	8.94 ± 0.9	30.78 ± 9.7	0.05	12.95 ± 3.2	25.78 ± 6.5	ns
	%				15.33 ± 2.6	10.5 ± 1.8	ns	24.34 ± 6	29.78 ± 5	ns	60.33 ± 5.7	59.72 ± 3.7	ns
C18 Sphinganine-1-phosphate	nM	302.82 ± 27.1	576.94 ± 127	ns	32.54 ± 7	51.8 ±10.2	ns	96.36 ± 8.2	99.43 ±15.3	ns	206.39 ± 17	410.29 ± 80.5	0.03
	%				8.23 ± 1.8	7.63 ± 1.2	ns	16.14 ± 1.2	9.56 ± 0.7	0.0008	75.63 ± 1.4	82.81 ± 1.6	0.006

Data are expressed as mean ± standard error. LPD=Lipoprotein Depleted. ns=not significant.

Supplementary Table 6. Sphingolipid levels in diet induced obese mice.

		Total Plasma			VLDL+LDL			HDL			LPD		
		Chow	HFD	p-value	Chow	HFD	p-value	Chow	HFD	p-value	Chow	HFD	p-value
C18 Sphingosine	nM	40.5 ± 8	30.29 ± 4.5	ns	10.09 ± 1.9	10.29 ± 2.6	ns	17.15 ± 3.4	28.94 ± 4.2	ns	36.73 ± 9.3	25.91 ± 4.2	ns
	%				15.55 ± 3.6	15.85 ± 4.4	ns	16.55 ± 3.8	28.51 ± 2.8	0.05	67.9 ± 5.7	55.65 ± 3	ns
C18 Sphinganine	nM	13.7 ± 2.4	10.47 ± 1.5	ns	2.93 ± 0.6	1.98 ± 0.2	ns	7.98 ± 0.3	8.37 ± 1.6	ns	9.62 ± 1.3	6.46 ± 0.9	ns
	%				13.43 ± 2.6	11.82 ± 1.4	ns	24.69 ± 2.7	32.22 ± 2.9	ns	61.88 ± 4.4	55.97 ± 3.7	ns
C18 Sphinganine-1-phosphate	nM	294.89 ± 7.2	439.75 ± 28.1	0.0003	31.19 ± 2.5	33.35 ± 2.6	ns	96.22 ± 6.1	133.53 ± 13.4	0.02	241.19 ± 8.3	274.07 ± 14.3	ns
	%				6.97 ± 0.7	6.39 ± 0.6	ns	14.2 ± 0.7	16.8 ± 1.1	ns	78.83 ± 1.1	76.81 ± 1.5	ns

Data are expressed as mean ± standard error. LPD=Lipoprotein Depleted. HFD = High Fat Diet. ns=not significant.

Supplementary Table 7. Sphingolipid levels in total plasma from control and L-FoxO1,3,4 mice after rescue of ApoM.

		Total Plasma				
		GFP		AdApoM		p-value
		Control	FoxO134	Control	FoxO134	
**C18 Sphingosine**	**nM**	63.26 ± 5.2	184.6 ± 97*	64.88 ± 2.6	84.44 ± 7.4*†	0.009
**C18 Sphinganine**	**nM**	38.28 ± 6.1	73.93 ± 21.1	35.89 ± 3.4	53.16 ± 2.6†	0.017
**C18 Sphinganine-1-phosphate**	**nM**	580.73 ± 67.6	728.56 ± 29.2	662.49 ± 38.1	842.85 ± 85.8	ns

Data are expressed as mean ± standard error. ns=not significant.
*p<0.05 vs Control-GFP. † p<0.05 vs Control-AdApoM, by Kruskal-Wallis 1-way ANOVA and Mann-Whitney U post hoc test.

Supplementary Table 8. Sphingolipid levels in lipoprotein fractions from control and L-FoxO1,3,4 mice after rescue of ApoM.

		non-HDL					HDL					LPD				
		GFP		AdApoM		p-value	GFP		AdApoM		p-value	GFP		AdApoM		p-value
		Control	FoxO134	Control	FoxO134		Control	FoxO134	Control	FoxO134		Control	FoxO134	Control	FoxO134	
C18 Sphingosine	nM	61.83 ± 39.4	32.92 ± 4	32.23 ± 11.2	34.34 ± 2.4	ns	56.58 ± 3.3	86.97 ± 10.9	60.58 ± 4.4	97.35 ± 17.1*‡	0.039	68.06 ± 5	107.24 ± 12.3*	54.61 ± 6.3	118.1 ± 10.5†§	0.004
	%	22.88 ± 9.7	13.47 ± 1.7	19.44 ± 4.1	12.80 ± 0.8	ns	20.87 ± 2.6	23.32 ± 2.5	27.58 ± 2.5	23.16 ± 1.8	ns	56.25 ± 7.4	63.21 ± 4	54.98 ± 3.2	64.04 ± 1.6	ns
C18 Sphinganine	nM	22.40 ± 11.4	17.1 ± 3.9	15.71 ± 3.6	21.36 ± 3.2	ns	32.78 ± 5.2	51.82 ± 5.2	42.01 ± 6.03	59.78 ± 11.81	ns	33.42 ± 4.7	55.82 ± 5.4*	36.48 ± 2.8	77.13 ± 12.3*§	0.004
	%	21.56 ± 9.2	12.55 ± 2.4	15.42 ± 2.4	12.47 ± 1.5	ns	23.81 ± 3.2	25.98 ± 2.9	28.22 ± 2.7	22.82 ± 3.1	ns	54.64 ± 7.5	61.47 ± 4.4	56.36 ± 3.1	64.7 ± 2.8	ns
C18 Sphinganine-1-phosphate	nM	47.1 ± 11.2	59.12 ± 8.9	50.89 ± 4.1	69.48 ± 7.23	ns	157.77 ± 24.9	126.06 ± 9	249.83 ± 24.5*	172.6 ± 25.9	0.029	416.8 ± 50.1	571.64 ± 58.9	444.56 ± 23.8	623.69 ± 53.3*‡	0.049
	%	7.23 ± 2.8	6.21 ± 1.1	5.81 ± 0.3	6.27 ± 0.4	ns	13.38 ± 1.1	8.8 ± 1*	18.88 ± 0.9†	10.67 ± 1.8§	0.002	79.39 ± 2.4	84.99 ± 2.1	75.3 ± 0.9	83.06 ± 2§	0.015

Data are expressed as mean ± standard error. LPD=Lipoprotein Depleted. ns=not significant. *p<0.05 vs Control-GFP. † p<0.01 vs Control-GFP. ‡ p<0.05 vs Control-AdApoM. § p<0.01 vs Control-AdApoM, by Kruskal-Wallis 1-way ANOVA and Mann-Whitney U post hoc test.

Supplementary Table 9. Primer sequences.

Gene	Direction	Sequence (5'-3')
36b4	Forward	AGATGCAGCAGATCCGCAT
36b4	Reverse	GTTCTTGCCCATCAGCACC
Apom	Forward	GTGCCCCGGAAGTGGACATACC
Apom	Reverse	AGCGGGCAGGCCTCTTGATTC
Foxo1	Forward	TCCAGTTCCTTCATTCTGCACT
Foxo1	Reverse	GCGTGCCCTACTTCAAGGATAA
G6pc	Forward	GTCTGGATTCTACCTGCTAC
G6pc	Reverse	AAAGACTTCTTGTGTGTCTGTC
Gck	Forward	CTGTTAGCAGGATGGCAGCTT
Gck	Reverse	TTTCCTGGAGAGATGCTGTGG
Igfbp1	Forward	AGATCGCCGACCTCAAGAAAT
Igfbp1	Reverse	CTCCAGAGACCCAGGGATTTT
Lipc	Forward	GACGGGAAGAACAAGATTGG
Lipc	Reverse	GGCATCATCAGGAGAAAGG
Scarb1	Forward	GGCTGCTGTTTGCTGCG
Scarb1	Reverse	GCTGCTTGATGAGGGAGGG
ChiP primer sequences		
Igfbp1	Forward	ATCTGGCTAGCAGCTTGCTGA
Igfbp1	Reverse	CCGTGTGCAGTGTTCAATGCT
G6pc	Forward	GCCTCTAGCACTGTCAAGCAG
G6pc	Reverse	TGTGCCTTGCCCCTGTTTTATATG
Apom #1	Forward	TCAGAGTCACTGGGTCATGC
Apom #1	Reverse	TGTTTTCCAACCCAAGCCTA
Apom #2	Forward	TTAAAGGGTCAAGGGTCGAG
Apom #2	Reverse	GAGTTGGTGCTCTGCAGTTG
Apom #3	Forward	TGCATGAGTCCCCACATCTA
Apom #3	Reverse	GTTCCAGGACAGCCAGAGAG
Apom #4	Forward	GGGAAATGTGGTGTCCTCTC
Apom #4	Reverse	TAAATGTGGGACTGGGGAAG

Supplementary Table 10. Antibodies used in flow cytometric analyses.

Antibodies	Cat #	Clone	Source	Fluorochrome
CD115	12-1152-82	AFS98	eBioscience	PE
CD11b	11-0112-85	M1/70	eBioscience	FITC
CD19	11-0193-85	eBio1D3	eBioscience	FITC
CD2	11-0021-85	RM2-5	eBioscience	FITC
CD3e	11-0031-85	145-2C11	eBioscience	FITC
CD4	11-0042-85	RM4-5	eBioscience	FITC
CD4	100531	RM4-5	Biolegend	Pacific Blue
CD45	557659	30-F11	BD Bioscience	APC-Cy7
CD45R	11-0452-85	RA3-6B2	eBioscience	FITC
CD8b	11-0083-85	eBioH35-17.2	eBioscience	FITC
CD8b	25-0083-82	eBioH35-17.2	eBioscience	PE-Cy7
cKit	105826	2B8	BioLegend	APC/Cy7
Gr1	553127	RB6-8C5	BD Bioscience	FITC
Gr1	552093	RB6-8C5	BD Bioscience	PerCP-Cy5.5
Sca1	108120	D7	BioLegend	Pacific Blue
Sca1	108114	D7	BioLegend	PE/Cy7
TCRβ	17-5961-83	H57-597	eBioscience	APC
TER119	11-5921-85	TER-119	eBioscience	FITC
